# A flexible, multifunctional, optoelectronic anticounterfeiting device from high-performance organic light-emitting paper

**DOI:** 10.1038/s41377-022-00760-5

**Published:** 2022-03-14

**Authors:** Teng Pan, Shihao Liu, Letian Zhang, Wenfa Xie, Cunjiang Yu

**Affiliations:** 1 State Key Laboratory of Integrated Optoelectronics, College of Electronics Science and Engineering, Jilin University, 130012 Changchun, China; 2 Department of Engineering Science and Mechanics, Department of Biomedical Engineering, Materials Research Institute, Pennsylvania State University, 16802 University Park, USA

**Keywords:** Organic LEDs, Optoelectronic devices and components

## Abstract

As a primary anticounterfeiting technology, most paper anticounterfeiting devices take advantage of photoresponsive behaviors of certain security materials or structures, thus featuring low-security threshold, which has been a critical global issue. To incorporate optoelectronic devices into existing anticounterfeiting technology suggests a feasible avenue to address this challenge. Here we report a high-performance organic light-emitting paper-based flexible anticounterfeiting (FAC) device with multiple stimuli-responsiveness, including light, electricity, and their combination. Without sacrificing the preexisted security information on the paper, we fabricate FAC device in a facile, low-cost yet high-fidelity fashion by integrating patterned electro-responsive and photo-responsive organic emitters onto paper substrates. By introducing optical microcavities, the FAC device shows considerable color shift upon different viewing angle and applied voltage, which is easily discernible by naked eyes. Notably, the FAC device is bendable, unclonable, and durable (a half-lifetime over 4000 hours at 100 cd m^−2^).

## Abstract (web summary)

Flexible optoelectronic anticounterfeiting device based on OLED paper can show different luminous states visible to the naked eye under light, electricity, and combined light and electricity stimulus.

## Graphical abstract

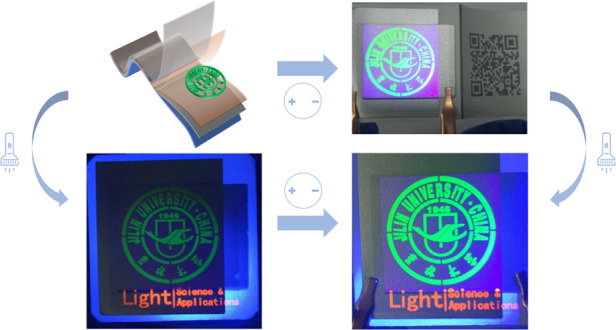

## Introduction

The past few decades have witnessed a fast-growing global issue in the spread of counterfeits, which are major threats to the economy, health, and environment^1–7^. Consequently, anticounterfeiting technologies, such as dithered patterns^8^, plasma tags^9^, security inks^10^, holograms^11^, watermarks^12^, and long-lag phosphors^13–17^, have been developed to prevent the potential infringements of counterfeiting. Primarily based on printing on flexible paper substrates, most anticounterfeiting technologies operate through photo-responsive behaviors of certain security materials or structures. The low-security threshold of the current anticounterfeiting technology that is authenticated by only photoresponsive is a long-standing challenge^18^. To develop a high-security anticounterfeiting system with multiple stimuli responsiveness is highly demanded.

Incorporating optoelectronic devices into existing paper-based photo-responsive anticounterfeiting technologies to achieve multiple stimuli responsiveness seems to be an ideal solution to improve the security level. Among various optoelectronic devices, organic light-emitting device (OLEDs) is an excellent alternative because it can simultaneously generate electro-responsive patterns governed by the anode–cathode overlapping area and photo-responsive patterns determined by the organic emitting area^19–22^. Nevertheless, the integration of OLED onto paper anticounterfeiting products to achieve high optoelectronic performances is technically challenging, and to the best of our knowledge, there is no relative report. Due to transport properties of organic semiconductors, organic functional layers must be thin (nanometer scale) enough to ensure efficient carrier transport. Owing to the paper’s porous and fibrous nature and its rough surface morphology, the devices usually lead to breakdown or short-circuit damages. Therefore, it has been a long-standing challenge to achieve high performance paper-based OLEDs^23–30^.

Here we report the development of a multiple stimuli-responsiveness flexible anticounterfeiting (FAC) device based on a high-performance organic light-emitting paper. Using commercially available paper with dip-coating treatment and successive multimaterial deposition, OLEDs were successively developed and their mechanical flexibility and the preprinted anticounterfeiting information of the paper was preserved without any loss. The organic light-emitting paper showed superior performances in terms of brightness, efficiency, and operating half-lifetime (~4000 h@100 cd m^−2^), which outperforms any existing counterparts^23–30^. Furthermore, a FAC device with multiple stimuli responsiveness was developed by integrating patterned electro-responsive and photo-responsive organic emitters onto a paper substrate. The FAC device provides multiple unclonable behaviors, including different patterns and colors in response to light, electricity, and combined light and electricity stimuli, and recognizable color shifts with different viewing angles and operating voltages. These unique multi-stimuli-responsive characteristics and device high performances render the anticounterfeiting device an unclonable and high-security-level feature.

## Results

### Preparation of organic light-emitting paper

**Fig. 1:**
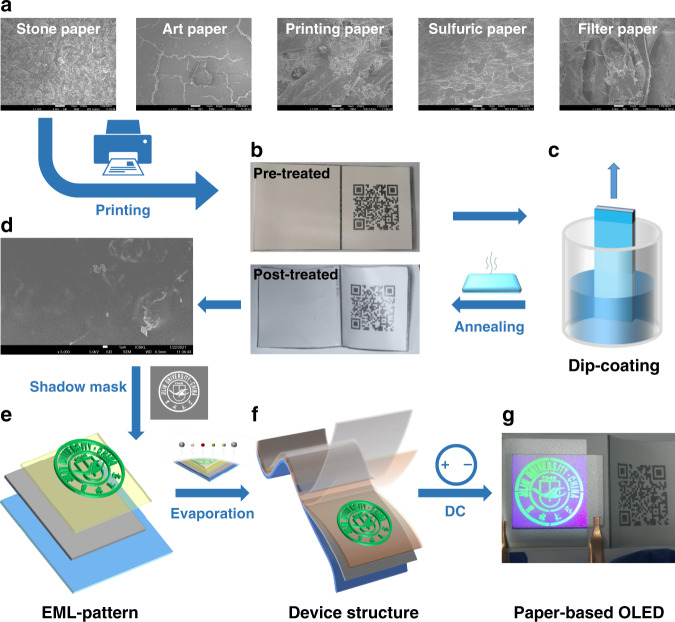
Organic light-emitting paper preparation. **a** SEM images of various types of commercially available paper. **b** Images of stone-paper substrates with QR code before and after dip-coating process. **c** Schematic diagram of the dip-coating process. **d** SEM image of stone paper treated by the dip-coating process. **e** Schematic diagram of patterned emitting layers. **f** Schematic diagram of the device structure of OLEDs. **g** Image of organic light-emitting paper at 5 V

First, five types of commercially available papers, including stone paper, art paper, printing paper, sulfuric paper, and filter paper were investigated. The scanning electron microscopical (SEM) images (bottom row) are shown in Fig. 1a. As can be seen in the images, all these different types of paper show a porous and rough morphology. Such morphology indicates that the paper cannot be used to prepare organic micro–nano devices, such that morphological modifications are needed. In addition, as seen in Figs. 1a and S1, the different types of paper show different microcosmic and microscopic morphologies. These morphologies are intrinsic features that can be used to identify the type of paper prepared by various papermaking processes and different material compositions. They also provide a way to identify or authenticate a paper-based document as the original.

To be environmentally friendly, the stone paper was chosen for further study. We printed a QR code (corresponding to our group website) on the stone paper by using a laser printer, as shown in Fig. 1b. To modify the morphology, the paper with the QR code was first treated by dip-coating in a solution of poly(methyl methacrylate) (PMMA). Details of the dip-coating process (Figs. 1c and S2) are included in the Experimental section. Upon treatment, a transparent PMMA can fill the porous paper (Fig. 1d) without sacrificing the paper’s intrinsic morphological features (see Figs. S1 and S3) and the preprinted QR code (bottom image of Fig. 1b). We then measured the surface morphology of the treated paper using an atomic force microscope (AFM). As shown in Fig. S4, the treated paper shows a very flat surface with a roughness of only 21.4 nm. The cross-section SEM of dip-coating paper is shown in Fig. S5. It can be seen that the thickness of the PMMA film formed by the dip-coating process is in the order of ten microns.

To prepare a green OLED on a treated-paper substrate (Fig. 1d), a patterned organic-emitting layer through a shadow mask (an institution logo, Figs. 1e and S6), and a sandwich structure (Fig. 1f) were successively deposited as described in Experimental section. Since the paper is opaque, we adopted a top-emitting structure for the OLED: Al (100 nm)/MoO_3_ (3 nm)/Di-[4-(N,N-di-p-tolyl-amino)-phenyl]cyclohexane (TAPC: 30 nm)/4″-tris(carbazol-9-yl)-trip-henylamine (TcTa: 5 nm)/4,4′-bis(carbazol-9-yl)biphenyl (CBP):10% tris (2-phenylpyridine) iridium(III) [Ir(ppy)_3_] (20 nm)/1,3,5-tris[(3-pyr-idyl)-phen-3-yl]benzene (TmPyPB: 50 nm)/LiF (0.5 nm)/Mg:Ag(15:1:1 nm/Ag (19 nm)). Upon applying 5 V voltage, the paper-based OLED can be turned on as shown in Fig. 1g. These results indicate that the morphological modification indeed offers the treated paper a feasible substrate for OLEDs.

### Performances of paper-based OLEDs

**Fig. 2:**
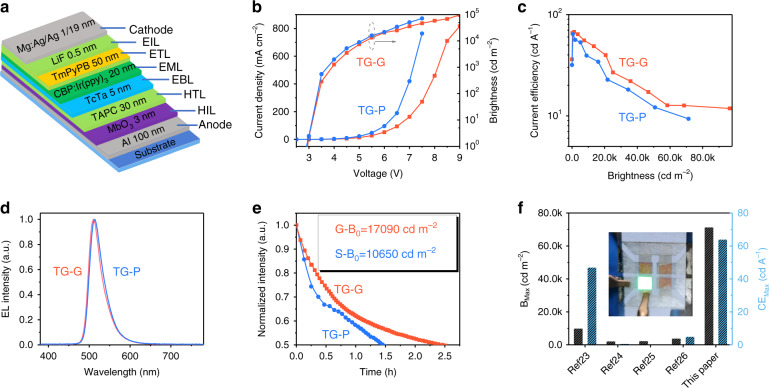
Device performances of “TG-P” and “TG-G”. **a** Schematic diagram of the device structure of OLEDs. **b** Current density–voltage–brightness, (**c**) current efficiency–brightness characteristics of green devices “TG-P” and “TG-G”. **d** Normalized spectra of green devices “TG-P” and “TG-G”. **e** Normalized intensity of devices “TG-P” and “TG-G” as a function of the operation time, with an initial brightness of *L*_0_ = 10,650 cd m^−2^ and *L*_0_ = 17,090 cd m^−2^ at constant driving current. **f** Maximum brightness and maximum current efficiency of paper-based OLEDs in reference papers and this work. The inset picture shows device “TG-P”

To investigate the performances of paper-based OLEDs, a green device with a fixed emitting area (3.3 × 3 mm^2^) was fabricated on the treated-paper substrate: this device is referred to as device “TG-P”. Details of the structure of device “TG-P” are shown in Fig. 2a and Table S1. For comparison, a glass-based OLED was also prepared using the same structure and is referred to as device “TG-G”. Figure 2b, c shows the current density–voltage–brightness and current efficiency–brightness characteristics of devices “TG-P” and “TG-G”. The EQE-brightness characteristics of “TG-P” and “TG-G” are shown in Fig. S7. The maximum brightness and maximum current efficiency of device “TG-P” are comparable to those of the glass-based OLED. Compared with glass substrate OLED, paper-based OLED has lower performance and faster J–V. The main reason is that paper substrate has higher surface roughness. After the dip-coating, the surface roughness of the paper substrate is greatly reduced, which has reached the requirements of the OLED flexible substrate. But compared with the glass substrate, the roughness is still relatively large, and there are some sharp peaks and holes on the surface. The surface morphology of paper substrate is directly transferred to the morphology of the organic layers, resulting in the significant leakage current of the device. Therefore, the paper-based OLED has a higher current and lower efficiency under the same voltage or the same brightness^31,32^. Figure 2d shows the normalized spectra of green devices “TG-P” and “TG-G”, which are similar.

Furthermore, the operating lifetime of devices “TG-P” and “TG-G” was also tested by operating the devices with a constant current of 5 mA. The results are shown in Fig. 2e. Using the acceleration coefficient of 1.7, the LT_50_ lifetime of device “TG-P” at 100 cd m^−2^ is estimated to be over 4000 hours^33^. The storage lifetime of paper-based OLEDs is also investigated (Fig. S8). Driven by a fixed current of 1 mA_,_ the luminance of the OLED maintains more than 90% of the initial value after storing for 10 days. More importantly, because the flexible thin-film encapsulation technologies are mature enough for displays, it can be considered that the storage lifetime and the operation lifetime of the paper-based OLEDs will meet the requirements of commercial applications.

The maximum brightness and current efficiency of device “TG-P” are 71346 cd m^−2^ and 64 cd A^−1^, respectively. When we used a treated art paper as the substrate, the maximum brightness and current efficiency of the green OLEDs can even reach 110000 cd m^−2^ and 90 cd A^−1^, respectively. To our knowledge, the brightness and current efficiency of our paper-based OLEDs are the highest values among all the paper-based OLEDs reported up to now. The performances of the reported paper-based OLEDs are summarized in Table S2 for reference. The maximum brightness and maximum current efficiency of paper-based OLEDs in reference papers and this work are shown in Fig. 2f. Moreover, top-emitting green OLEDs with the other types of commecial paper substrate treated by the dip-coating process are also prepared, and their performances are summarized in Table S3. It can be seen that the dip-coating process can also efficiently improve performances of devices with the other types of commercial paper substrate.

**Fig. 3:**
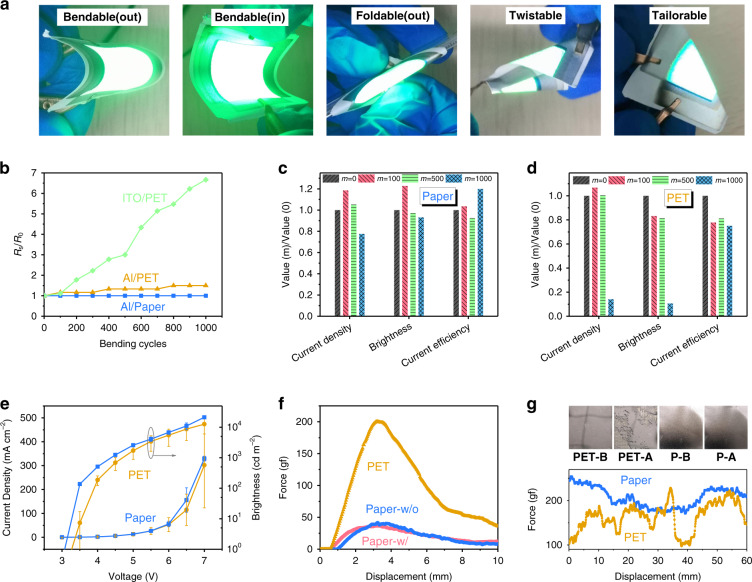
Mechanical properties of substrates and devices. **a** Images of paper-based green OLEDs after being bent, folded, twisted, and cut in half. **b** Current density–voltage–brightness of “TG-P” and “TG-PET” at different bending cycles and the variance. **c**, **d** Variations of current density, brightness, and current efficiency of devices (**c**) “TG-P” and (**d**) “TG-PET” before and after bending, and the value (m) represents the corresponding value of the device characteristics after bending m times. **e** Sheet resistance of the Al electrode based on stone paper, PET, and PET/ITO at different bending cycles. **f** Force-deflection characteristics of stone-paper substrates with (Paper-w/) and without (Paper-w/o) dip-coating treatments and PET substrates. **g** (upper row) Top-view images of the Al electrodes before and after peel tests, and (bottom row) peeling force–displacement characteristics of Al electrodes (100 nm) coated on paper and PET substrates

The paper-based OLEDs also show excellent mechanical flexibility. As Fig. 3a, movie S1, and movie S2 show, the paper-based OLEDs are bendable, foldable, twistable, and tailorable, such characteristics are difficult to achieve in OLED with glass or plastic substrate. We then measured the performances of device “TG-P” after continuously bending 100, 500, and 1000 times at a radius of 8 mm. For comparison, a PET-based device “TG-PET” with the same device structure was also investigated. The characteristics of devices “TG-P” and “TG-PET” after bending are shown in Figs. S9a, 6b, respectively. The variations of the current density, brightness, and current efficiency of devices “TG-P” and “TG-PET” at 5 V are summarized in Fig. 3b–d. It can be seen that device “TG-P” shows slight variations in performances after continuously bending 100, 500, and 1000 times. In contrast, the performances of device “TG-PET” dropped sharply as the number of bending cycles increased. After bending 1000 times, parts of device “TG-PET” could not be turned on (Fig. S10). It should be noted that the bending tests mainly reduce the current densities and the brightness, rather than the current efficiencies. It indicates that the organic-function layers would still work normally with a decreased current density under the same bias voltage after bending. The decreased current densities should be attributed to the increased sheet resistances of the electrodes due to the bending cracks. Figure S11 shows the microscopic images of device “TG-P” and the normally operating area of device “TG-PET”. It can be seen that after continuous bending, device “TG-PET” shows a large number of black cracks and dots, while device “TG-P” shows only some small cracks. As noted above, the paper-based OLEDs perform excellent mechanical flexibility.

The total thickness (hundreds of nanometers) of various functional layers is far below that (hundreds of micrometers) of the substrate (Table S4). As a result, the mechanical properties of the substrates are considered to be the main factors that determine the flexibility of the whole device. The sheet resistance of the electrode before and after bending was measured, and the results are shown in Fig. 3e. The force-deflection characteristics of the PET substrate and the stone-paper substrates with (Paper-w/) and without (Paper-w/o) dip-coating treatments are measured by a three-point bending flexural test (Fig. S12). The measured force-deflection characteristics are shown in Fig. 3f. The values of flexural modulus (E_f_) and bending strength (R) of these substrates can then be extracted from the force-deflection characteristics^34–36^. As shown in Table S5, the values of E_f_ and R of the paper substrates are only one-quarter of those of the PET substrate. It is well known that the higher the E_f_ and the R, the stiffer the material, while the lower the E_f_ and the R, the more flexible the material. Thus, the results (Fig. 3f) indicate that the paper substrates have much better flexural property than the PET substrate with the same thickness. As a result, the stress generated by the stone-paper substrate is smaller under the same bending state. During the bending processes, the higher stress generated by the PET substrate is considered to cause more serious damage to the device, especially the electrodes (see the scratches in Figs. S10, S11 and the reduced current densities in Fig. 3c, d). Furthermore, the enhanced adhesion between the Al electrode and the paper substrate is also considered to improve the flexural property of our paper-based OLEDs. We conducted the peel-adhesion tests (Fig. S13) to compare the adhesion of the Al electrode on the paper substrate and the PET substrate. A 3 M adhesive tape (scotch) was tightly pasted on the Al films (100 nm) coated on the paper and the PET substrates. We then measured the peeling force of removing the 3 M adhesive tape with the displacement changes. The results are shown in Fig. 3g. It shows that a much smaller peel force is required to remove the 3 M adhesive tape on the PET substrate. Besides, the images of the Al electrode before and after the peeling adhesion tests are shown in the upper row of Fig. 3g. Here, PET- and P- respectively represent the PET and the paper substrates, while -B and -A represent the cases before and after the peeling-adhesion tests, respectively. It can be observed that the Al electrode on the PET substrate (PET-A) is not intact after tests, while the Al electrode on the paper substrate (P–A) is. We can thus conclude that the adhesion between the paper substrate and the Al electrode is much stronger than that between the Al electrode and the PET substrate. This difference is attributed to the higher roughness (Fig. S4) of the paper surface at the nanoscale^37–39^.

### Optoelectronic FAC device with multiple stimuli responsiveness

**Fig. 4:**
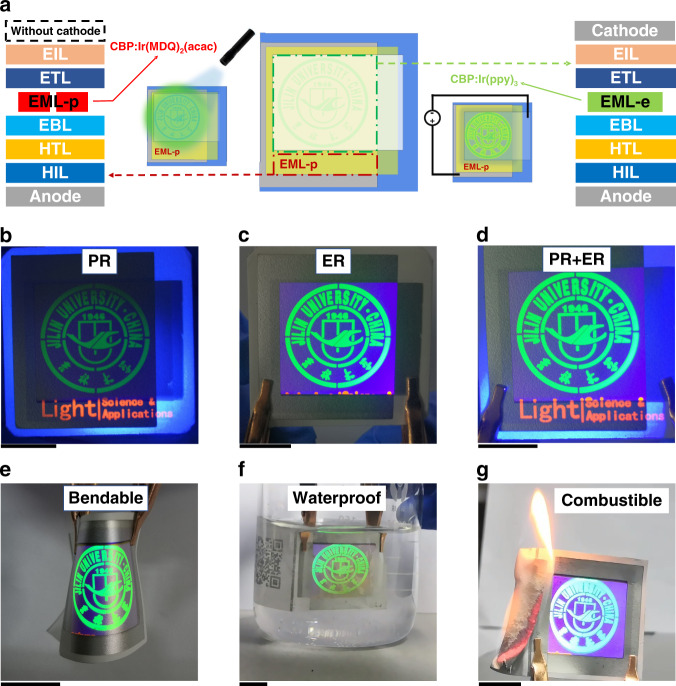
Anticounterfeiting device with multiple stimuli responsiveness. **a** Schematic diagrams of the working mechanism of the proposed flexible anticounterfeiting device. **b** Photoresponse (PR) of the FAC device under 365 nm light stimuli, **c** electro-response (ER) of the FAC device under 5 V, **d** PR and ER of the FAC device under light and electricity stimuli. Images of the FAC device in response to electricity stimuli, **e** after being bended, **f** after being put into deionized water, and **g** after being set on fire. Black scale bar of each figure represents the length of 1 cm

As aforementioned, the compatibility between organic electronics and paper substrates can be addressed by paper morphological modifications. Based on this, we designed and achieved a FAC device with multiple stimuli responsiveness, as shown in Fig. 4a. The FAC device was achieved by integrating patterned electro-responsive and photo-responsive organic emitters onto a polymer-coated porous paper substrate. In this work, two patterned emitters EML-e and EML-p were prepared by combining vacuum thermal evaporation and patterned metal masks (Fig. S6a, b). The pattern of the EML-e was designed as our university logo (Fig. S6a), while that of the EML-p was designed as English words “Light: Science & Applications”.

Both the emitters were deposited on the paper substrate coated with a large-area reflective Al anode (purple area in Fig. 4a) and hole-transport layers (MoO_3_/TAPC/TCTA). After the deposition of the two emitters, the large-area electron transport layer (TmPyPB) and the semitransparent cathode (LiF/Mg:Ag/Ag) were sequentially deposited on the paper substrate. The difference between the two emitters is that the EML-e is covered by the cathode (the semitransparent rectangle of the middle schematic diagram of Fig. 4a), while the EML-p is not.

Figure 4b–d shows the images of the FAC device under light, electricity, and combined light and electricity stimuli, respectively. Under excitation of a 365 nm ultraviolet (UV) light, a green university logo and an orange “Light: Science & Applications” can be clearly observed (Fig. 4b). The green logo and the orange words exactly correspond to the photoluminescent (PL) emission from the EML-e and the EML-p, respectively. When a voltage of 5 V was applied, a green university logo with a violet background was observed at the anode–cathode overlapping area (Fig. 4c). The green logo and the violet background correspond to the electroluminescent (EL) emission from the EML-e and the TCTA, respectively. The violet background was not observed under the excitation of the 365 nm UV light. This is due to the PL emission of the transport materials that can only be excited by a UV light with a much shorter wavelength^40–42^. Nevertheless, carrier injections from the electrodes can directly form excitons at the interfaces between the TCTA and the TmPyPB, resulting in the EL emission from the TCTA with a smaller singlet-energy level. Because the TmPyPB and TAPC/TCTA respectively have a deep HOMO and LUMO level (Fig. S14), the formed singlet excitons will be efferently formed at the TCTA/TmPyPB interface without producing a high current leakage. This property also provides the FAC device a third behavior in response to a light and electricity stimulus. Excited by a 365 nm UV light and a bias voltage of 5 V simultaneously, a green university logo with a violet background and an orange “Light: Science & Applications” are observed as shown in Fig. 4d. In fact, the EL emission of the EML-e is much brighter than its PL emission (movie S3). These triple stimuli responsiveness of the FAC device will be much easier for human eyes to recognize. Besides, it can be noted that the paper’s intrinsic feature (Fig. 4c, d) is also kept after integration with the FAC device. These results demonstrate the compatibility between the FAC device and the current paper-based anticounterfeiting technologies.

The FAC device also shows several properties with high technical barrier for counterfeit manufacture. First, the FAC device has excellent flexibility (Fig. 4e) such that it can be attached to some curved surfaces for anti-counterfeiting. Second, the FAC device shows an excellent waterproof property. After coating the top of the semitransparent cathode with a UV-cured adhesive layer, the paper-based FAC device can still work normally in deionized water (Fig. 4f, movies S4, S5). We designed an experiment (Fig. S15) to clarify the issues of WVPR and OTR for the dip-coating paper in our work. The test results are shown in Fig. S16. It can be found that the treated paper shows much better oxygen/water-barrier abilities than the commercial PET. In fact, the current thin-film encapsulation technologies can also be used to further enhance the oxygen/water-barrier abilities without sacrificing the anticounterfeiting information stored in the paper (see Figs. S1, S3). Third, the paper-based FAC device is combustible (Fig. 4g and Movie S6). Since hydrocarbons compose the combustible paper and the PMMA, the complete combustion of the paper-based FAC device will mostly produce carbon dioxide and water. It is thus eco-friendly for wide-scale use in daily life. Finally, we also conducted the tests against scratches (Fig. S17) and shear stress (Fig. S18) of the paper substrates. The results show that the properties against scratches and shear stress of the pristine paper have been also kept by the treated paper.

**Fig. 5:**
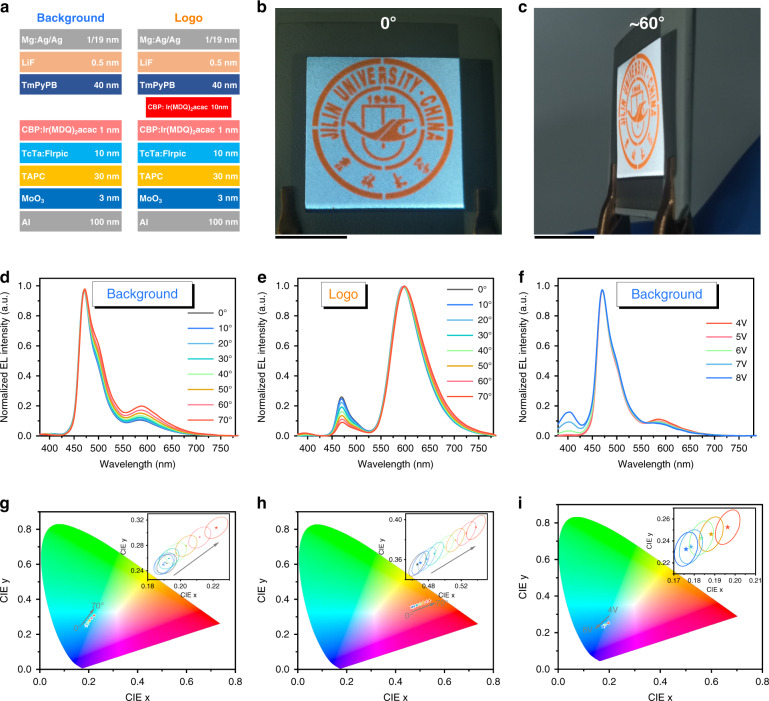
Color-shift characteristics of white and orange FAC device. **a** Details of the structures of the background and logo areas of the white and orange FAC device. **b** Front-view image of the white and orange FAC device in response to electricity stimuli (5 V), and (**c**) its image at a viewing angle of about 60°. **d** Normalized spectra of the device “background” at different viewing angles. **e** Normalized spectra of the device “logo” at different viewing angles. **f** Normalized spectra of the device “background” at different bias voltages. **g** CIE coordinates and their 5-step MacAdam ellipses of the device “background” at different viewing angles. **h** CIE coordinates and their 5-step MacAdam ellipses of the device “logo” at different viewing angles. **i** CIE coordinates and their 5-step MacAdam ellipses of the device “background” at different bias voltages. Black scale bar of each figure represents the length of 1 cm

In addition to the above properties, the cathode–anode overlapping area of the FAC device shows considerable color shifts with angle and voltage changes. To investigate the color shifts, we prepared a bilayer EML-e, including a white emitter and an orange emitter. The device structure of the white and orange FAC device is shown in Fig. 5a. The white emitter with a structure of TCTA: 20 wt% Bis (3,5-difluoro-2-(2-pyridyl) phenyl-(2-carboxypyridyl)) iridium (III) (Firpic) (10 nm)/CBP: 3 wt% iridium(III) bis (2-methyldibenzo[f, h]quinoxaline)-acetylacetonate [Ir(MDQ)_2_(acac)] (1 nm) has a pattern of our university logo. Figure 5b, c respectively shows the images of 0^o^ and 60^o^ viewing angles when a bias voltage of 5 V was applied on the FAC device. As shown in Movie S7, the increase of the viewing angle leads to a substantial redshift in the emission of both the background and the logo areas. This is considered to be due to the microcavity effect formed by the highly reflective anode and the reflective/semitransparent cathode. In fact, according to the classical electromagnetic theory, the emission spectrum of top-emitting OLED should show a blue shift. However, compared with the conventional top-emitting devices, the top-emitting OLEDs presented in Fig. 5d, e have much shorter cavity lengths. It thus leads to a resonant wavelength much below the wavelengths of blue emission from Firpic and orange emission from Ir(MDQ)_2_(acac). As a result, we consider that the classical electromagnetic theory may be not suitable for this case (see the unchanged emission wavelengths of the spectra), but the Purcell effect of a quantum emitter (see Note S1) should play a dominant role in spectral variations (see the relative contributions of blue and red emissions). Besides, a substantial blueshift is also observed with the increase of the bias voltage (Fig. S19 and Movie S8). The color shifts can provide a feature with a high technical threshold for the FAC device.

To quantitatively investigate the color shifts, we prepared two OLEDs with a small area of ~10 mm^2^. One has the same structure as the background area described above and is referred to as device “background”, while the other has the same structure as the logo areas described above and is referred to as device “logo”. Figure 5d, e shows the normalized EL spectra of devices “background” and “logo” at different viewing angles, respectively. It can be seen that the relative contributions of the orange emission of devices “background” and “logo” increase as the viewing angle increases. Figure 5f shows the normalized EL spectra of device “background” at different bias voltages. On the contrary, the relative contributions of the orange emission of device “background” decrease as the bias voltage increases.

Chromaticity coordinates in the Commission Internationale de L’Eclairage (CIE) 1931 color space are defined quantitative relations between the emission spectrum and physiologically perceived colors of human eyes. The chromaticity coordinates of devices “background” and “logo” at different viewing angles are shown in Fig. 5g, h, respectively. The data suggest that the chromaticity coordinates of devices “background” and “logo” change as the viewing angle increases. A MacAdam ellipse is a region on a chromaticity diagram that contains all colors that are indistinguishable, to the average human eye, from the color at the center of the ellipse^43^. Chromaticity coordinates beyond the ellipse therefore represent a color that is distinguishable from the color at the center of the ellipse. Five-step MacAdam ellipses of the chromaticity coordinates of devices “background” and “logo” at different viewing angles are calculated and shown in the insets of Fig. 5g, h, respectively. Apparently, with the variation of viewing angle, color shifts can be easily recognized by human eyes. Figure 5i shows the chromaticity coordinates of device “background” at different bias voltages and their corresponding five-step MacAdam ellipses. This result supports the conclusion that distinguishable color shifts occur with the variation of the bias voltage.

In fact, photo-responsive, chemical-responsive, and mechanical-responsive patterns have been developed for anticounterfeiting technology. Compared with the current anticounterfeiting technologies, the multiple stimuli responsiveness of our FAC device would show much more advantages in terms of reliability, practicality, identifiability, and convenience of stimulus (see Table 1 and Movie S9)^44^.

**Table 1:** The advantageous and disadvantageous properties of this work and the current anticounterfeiting technologies. The “AC” means anticounterfeiting

Types	Reliability	Practicality	Identifiability	Complexity	Convenience of stimulus
Electro responsive AC	High	High	High	Medium	Easy@(power bank)
Photo responsive AC	Low	Medium	Medium	Low	Difficult@(Multiple light sources)
Chemical responsive AC	High	Low	Low	Medium	Difficult@(Chemical reagent)
Mechanicalr responsive AC	Medium	High	Low	Medium	Easy@(Manpower)
Heat responsive AC	Medium	Low	Low	Medium	Medium@(Heating source)

## Discussion

We demonstrated a flexible, high-performance organic light-emitting paper-based anticounterfeiting device. Through the paper-surface morphology modification, the treated paper is proven to be a compatible and feasible substrate for OLEDs. Owing to the treatment, the yielded OLEDs with record-high brightness, efficiency, and operating lifetime are achieved. We further demonstrated for the first time a multi-material-architectured FAC device with multiple stimuli-responsive behaviors, whose responsive patterns are determined by the planar structural designs. Different light-emitting patterns and colors in response to photo-, electro- and photo-/electro-stimuli have been achieved. Besides the unique multiple stimuli responsiveness, the FAC device is also eco-friendly, waterproof, and able to shift color with different viewing angles and bias voltages, which collectively raises a significantly high technical barrier for counterfeit manufacture. Such flexible, high-performance organic light-emitting paper-based FAC device sheds light on the next-generational high-security level anticounterfeiting technologies and holds promise in many conventional and emerging applications, such as anticounterfeiting label or packaging of valuable products, anticounterfeiting of certificates, calligraphy, and painting works. To incorporate optoelectronic devices into existing anticounterfeiting technology, as illustrated in this study, shed light on solutions toward the outstanding issue of counterfeits.

## Materials and methods

### Materials

The stone-paper substrate was purchased from Shenzhen Stone Paper Enterprise. MoO_3_ and LiF powder, small molecular organic materials, such as, 4,4′-bis(carbazol-9-yl)biphenyl (CBP), di-[4-(N,N-di-p-tolyl-amino)-phenyl]cyclohexane (TAPC) 4,4′,4″-tris(carbazol-9-yl)-trip-henylamine (TCTA), 1,3,5-tris[(3-pyr-idyl)-phen-3-yl]benzene (TmPyPB), bis(3,5-difluoro-2-(2-pyridyl)phenyl-(2-carboxypyridyl))iridium(III) (FIrpic), and iridium(III) bis(2-methyldibenzo[f, h]quinoxaline)- acetylacetonate [Ir(MDQ)_2_(acac)], were purchased from Luminescence Technology Corp. While tris (2-phenylpyridine) iridium(III) [Ir(ppy)_3_] was obtained from Xi’an p-OLED.

### Dip-coating process

The paper substrate was treated by dip-coating in the butyl acetate solution containing 7 wt% polymethyl methacrylate (PMMA). The process is as follows: first, the paper substrate was immersed in the PMMA solution for 20 min. Subsequently, the substrate was withdrawn in the vertical direction. As the withdrawal speed increased, the thickness of the buffer layer and the surface roughness increased. According to the requirements of this experiment, we set the withdrawal speed to 3 cm s^−1^. The whole process is carried out under ambient temperature (25 °C) and humidity (35%). Suspend the substrate with clips and place it in air at room temperature (25 °C) and humidity (35%) for 20 min and the solvent in the liquid film was partially evaporated. The above procedure should be repeated twice. Finally, the paper substrate was put in an oven and annealed at 70 °C for 20 min to completely evaporate the solvent in the liquid film. The resulting substrate can be used as a flexible substrate for OLEDs.

### Device fabrication

Various structures of OLEDs were fabricated on different substrates, and all the device structures are shown in Table S1. All of the paper substrates were treated by the dip-coating process. The ITO-coated glass substrates were cleaned by Decon 90, and ultrasonically cleaned in deionized water three times for 5 min. Then the glass was dried at 120 °C for 20 min. All of the types of substrates were put in a vacuum evaporator. Finally, electrode materials and organic functional layers were thermal deposited sequentially on the substrates under vacuum of 6.0 × 10^−4 ^Pa.

### Characterization setup

Voltage–current–brightness characteristics and electroluminescence spectra of devices without any protection were measured by Goniophotometric Measurement System (GP500, Otsuka Electronics Co. Osaka, Japan) in air at room temperature simultaneously. The surface morphologies of the paper substrates were characterized by a tapping-mode atomic force microscope (AFM) (Dimension Icon, Bruker Co.) and field-emission scanning electron microscope (FESEM). The flexural modulus E_f_ and bending strength R were tested with a mechanical analysis testing machine (FT-8000D, Suzhou F-Tom testing equipment Co., Ltd, China).

## Supplementary information

SUPPLEMENTAL MATERIAL

Movie S1

Movie S2

Movie S3

Movie S4

Movie S5

Movie S6

Movie S7

Movie S8

Movie S9
