# Supplementary material for: A flexible, multifunctional, optoelectronic anticounterfeiting device from high-performance organic light-emitting paper
Source: Light Sci Appl. 2022 Mar 14;11:59. doi: 10.1038/s41377-022-00760-5 (PMC8921225; doi:10.1038/s41377-022-00760-5)
Supplement: Supplementary file 1 — SUPPLEMENTAL MATERIAL [file 41377_2022_760_MOESM1_ESM.docx]

Supplementary Information for

A flexible, multifunctional, optoelectronic anti-counterfeiting device from high-performance organic light-emitting paper

*Teng Pan* ^1†^, *Shihao Liu*^1†^, *Letian Zhang^1^*, *Wenfa Xie*^1^, *Cunjiang Yu*^2^

^1^State Key Laboratory of Integrated Optoelectronics, College of Electronics Science and Engineering, Jilin University, 130012 Changchun, China

^2^Department of Engineering Science and Mechanics, Department of Biomedical Engineering, Materials Research Institute, Pennsylvania State University, 16802 University Park, USA

Correspondence: Wenfa Xie (xiewf@jlu.edu.cn) or Cunjiang Yu (cmy5358@psu.edu)

^†^These authors contributed equally to this work.


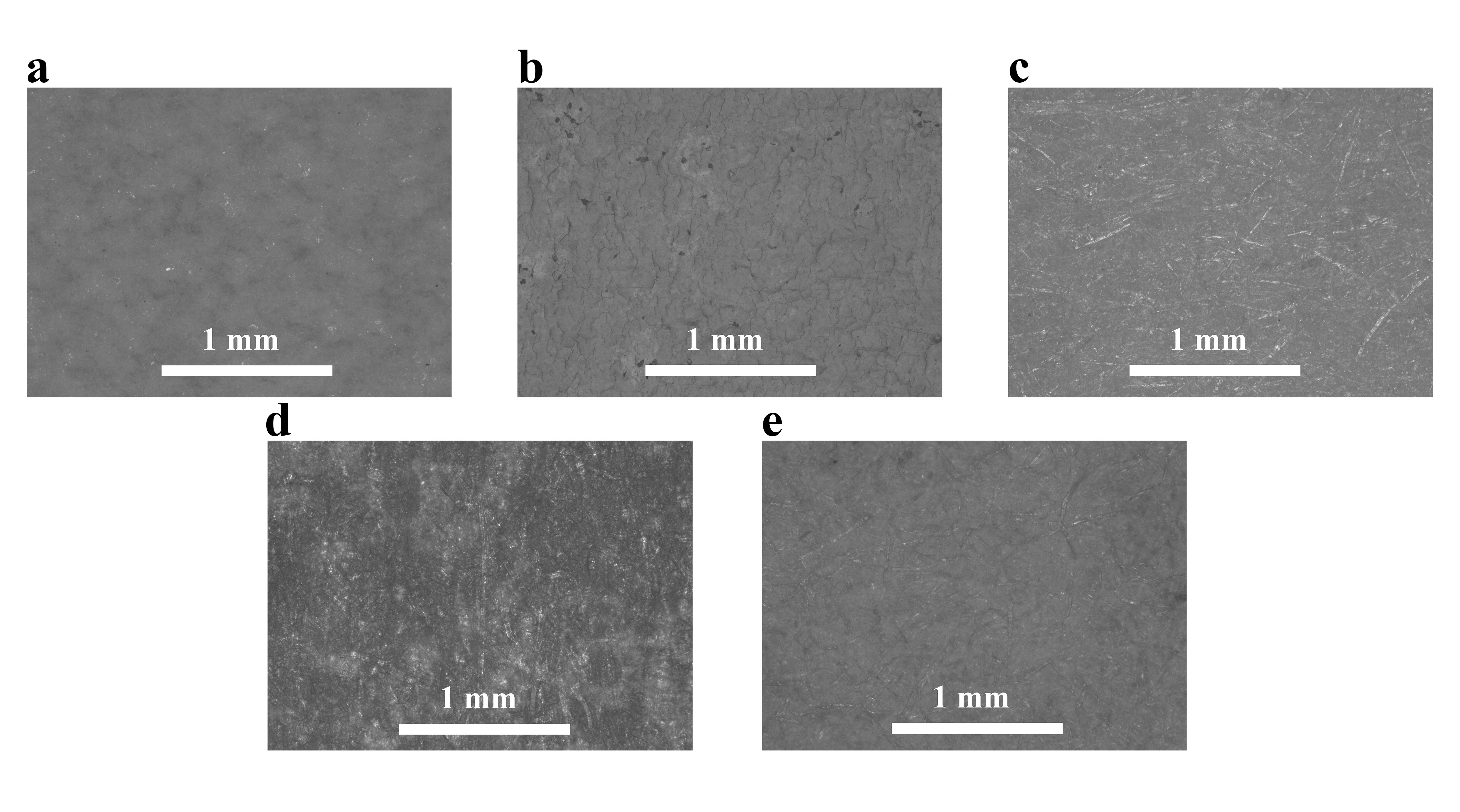


Fig S1. Optical microscope images of raw paper. a) stone paper, b) art paper, c) print paper, d) sulfuric paper, e) filter paper.


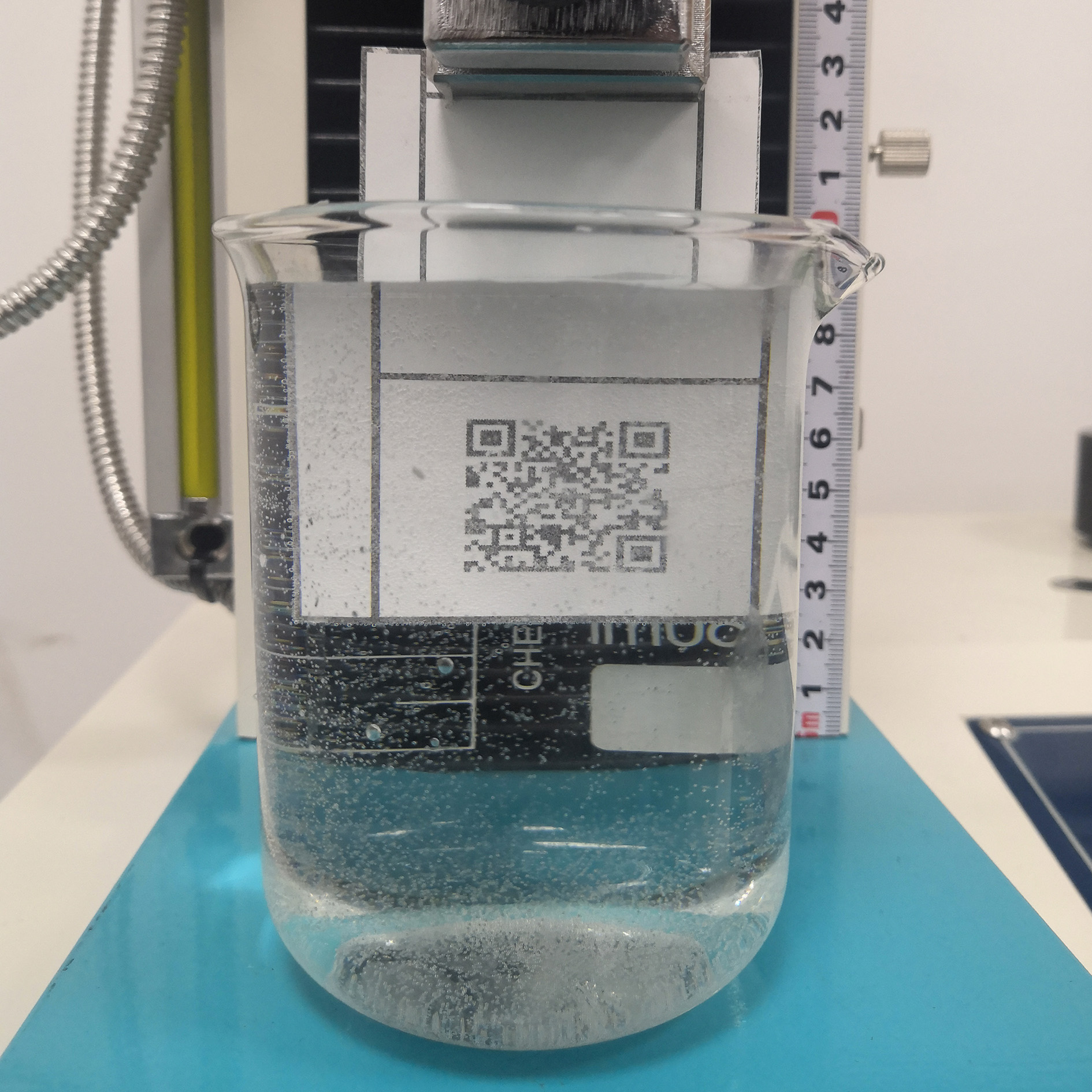


Fig S2. Photo of the dip-coating setup.


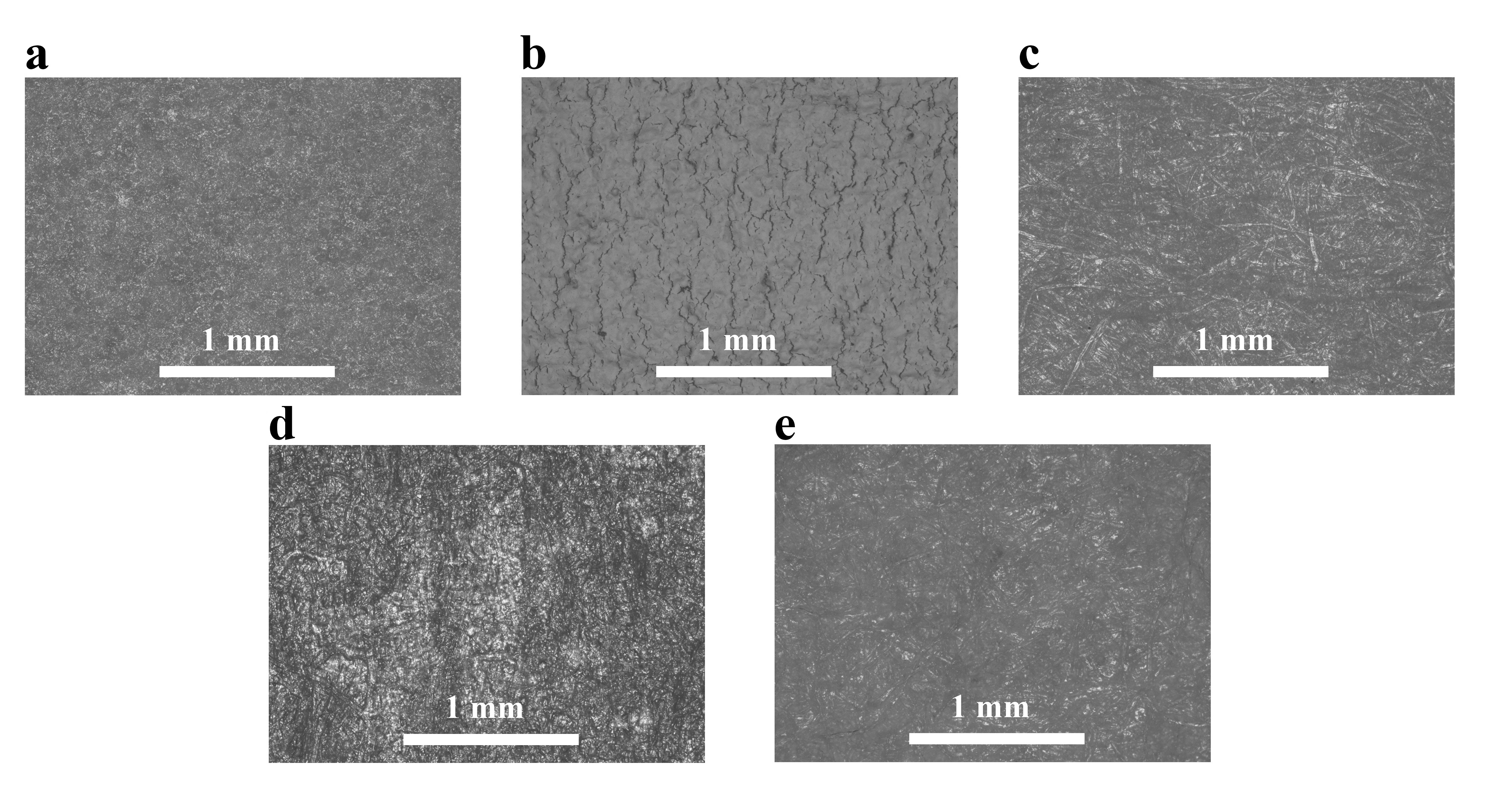


Fig S3. Optical microscope images of different papers after dip-coating process. a) stone paper, b) art paper, c) print paper, d) sulfuric paper, e) filter paper.


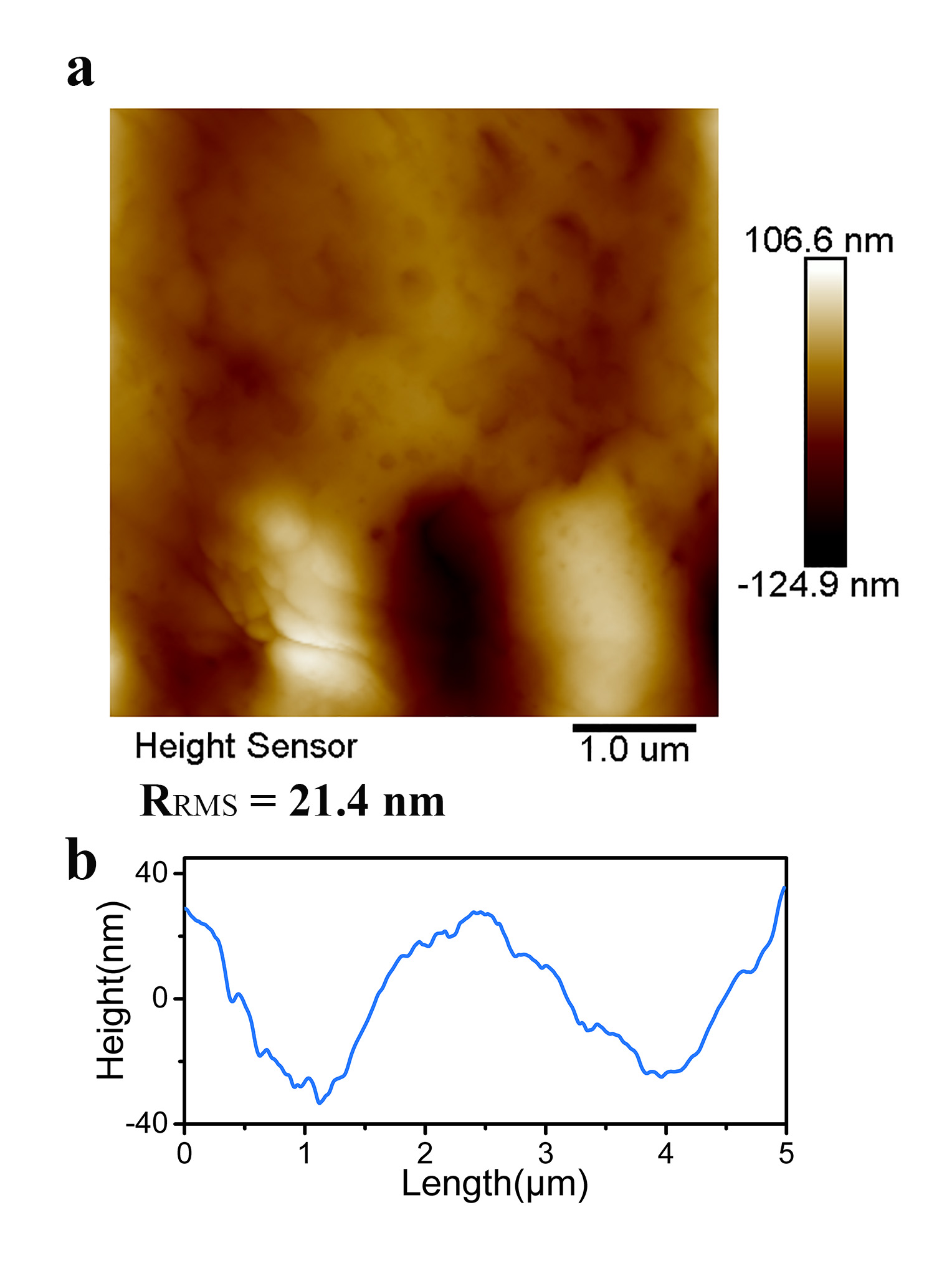


**Fig S4.** AFM a) height image and b) cross-section height image of the treated stone paper.


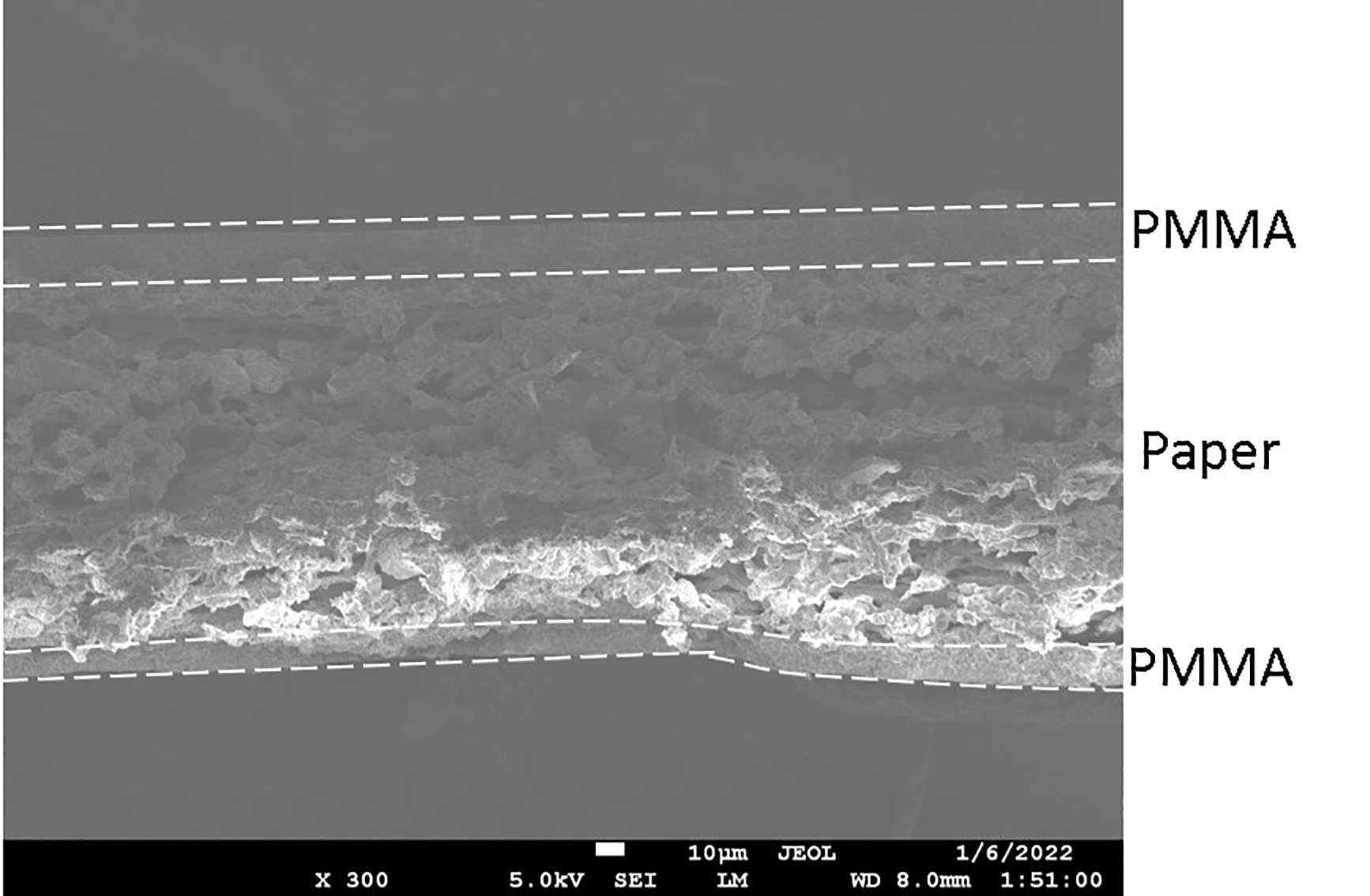


**Fig S5.** The cross section SEM of dip-coating paper.


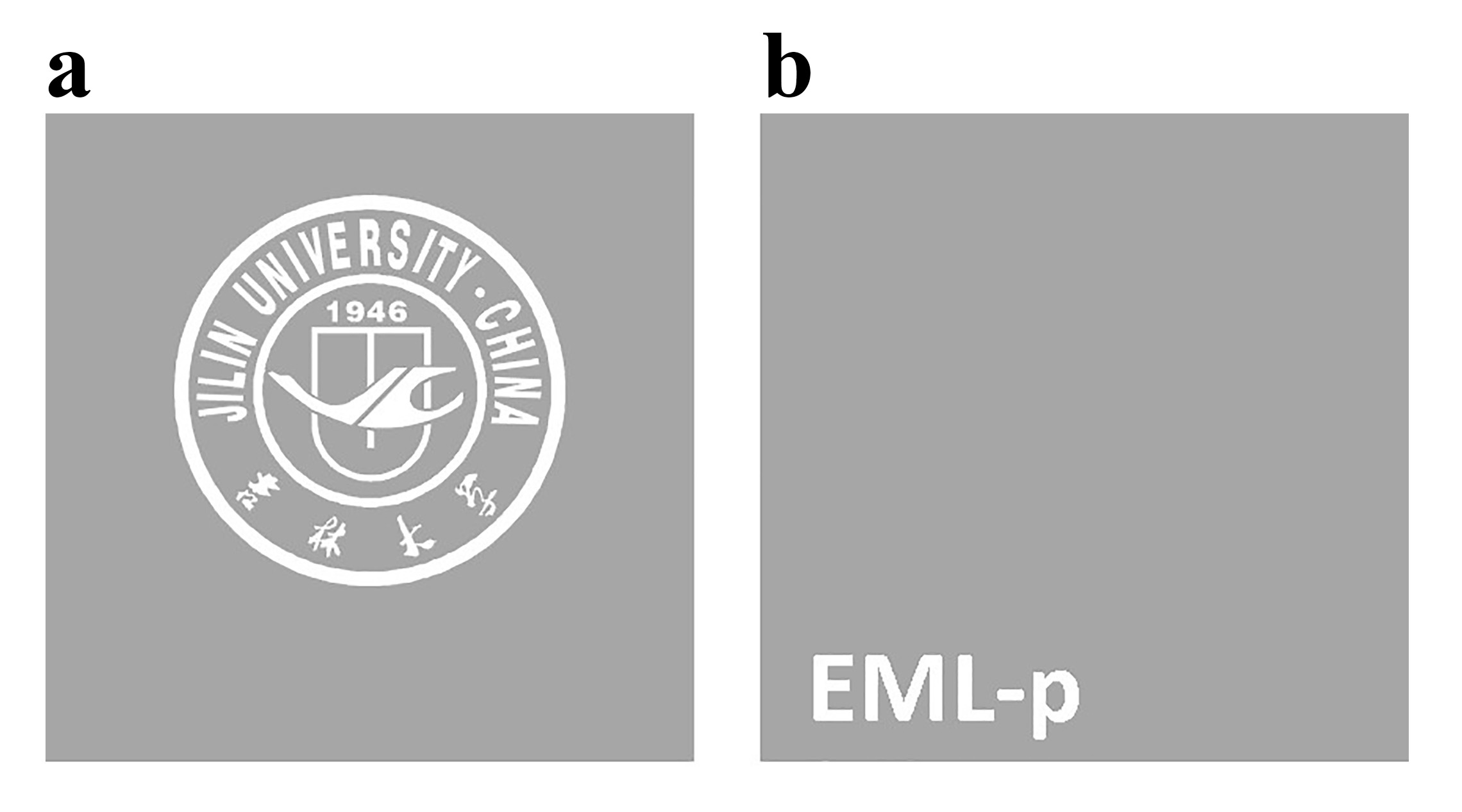


**Fig S6.** Photos of shadow masks used for preparing patterned emitting layers.


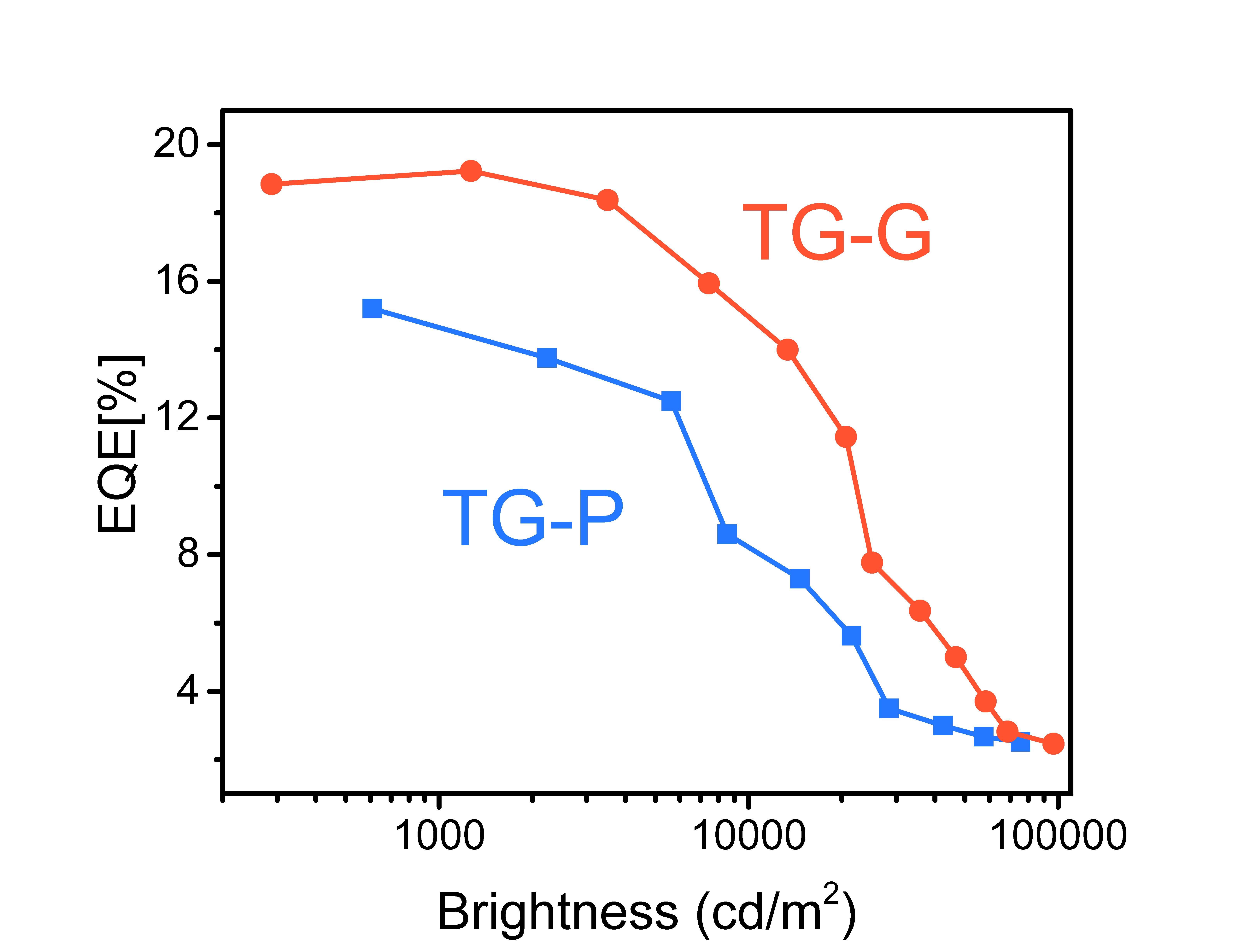


**Fig S7.** EQE-brightness characteristics of devices TG-P and TG-G.


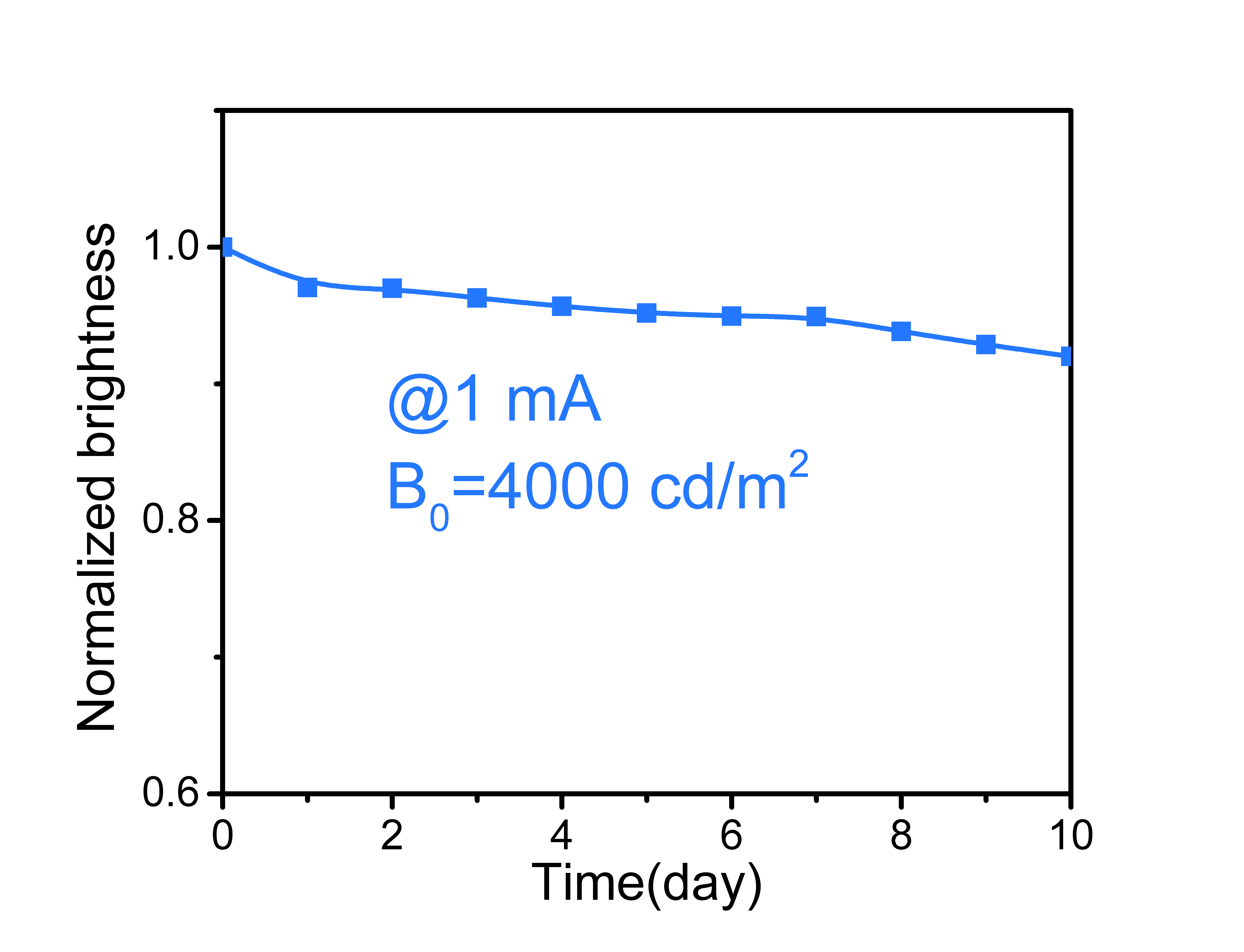


**Fig S8.** Normalized brightness of paper-based OLED driven by the current of 1 mA after storing in the glovebox for several days.


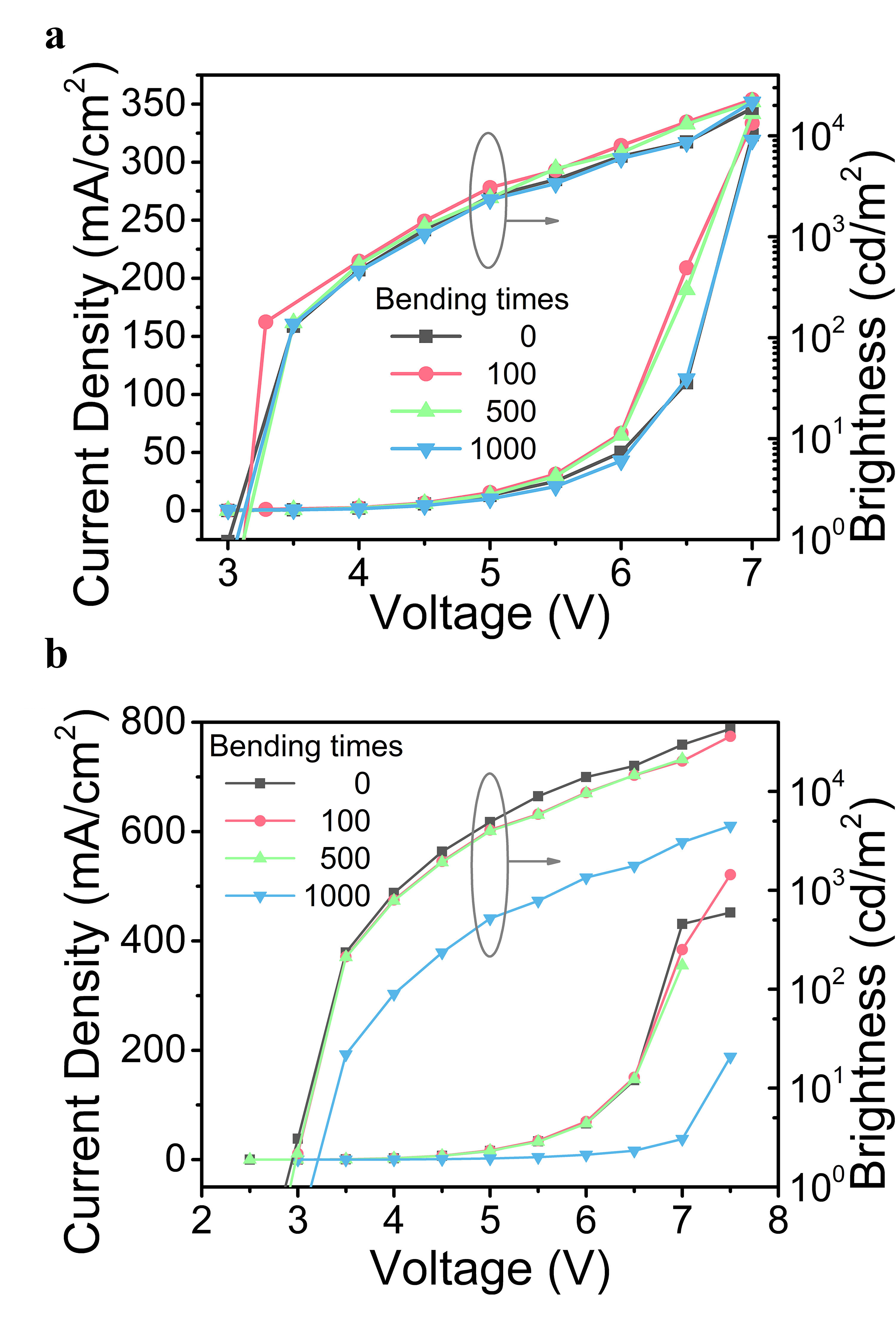


**Fig S9**. Current density-voltage-brightness characteristics of a) paper-based OLEDs and b) PET-based OLEDs before and after bending tests.


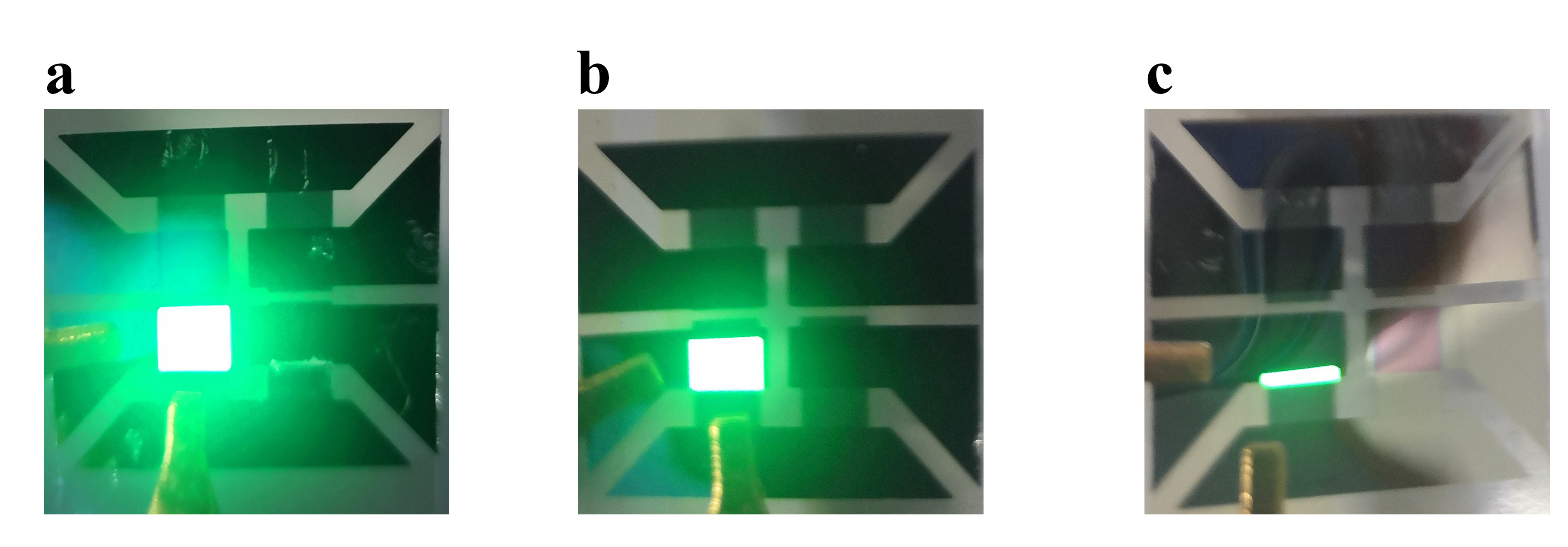


**Fig S10**. Optical images of PET-based OLED before and after bending. a) without bending, b) after bending 500 times, c) after bending 1000 times.


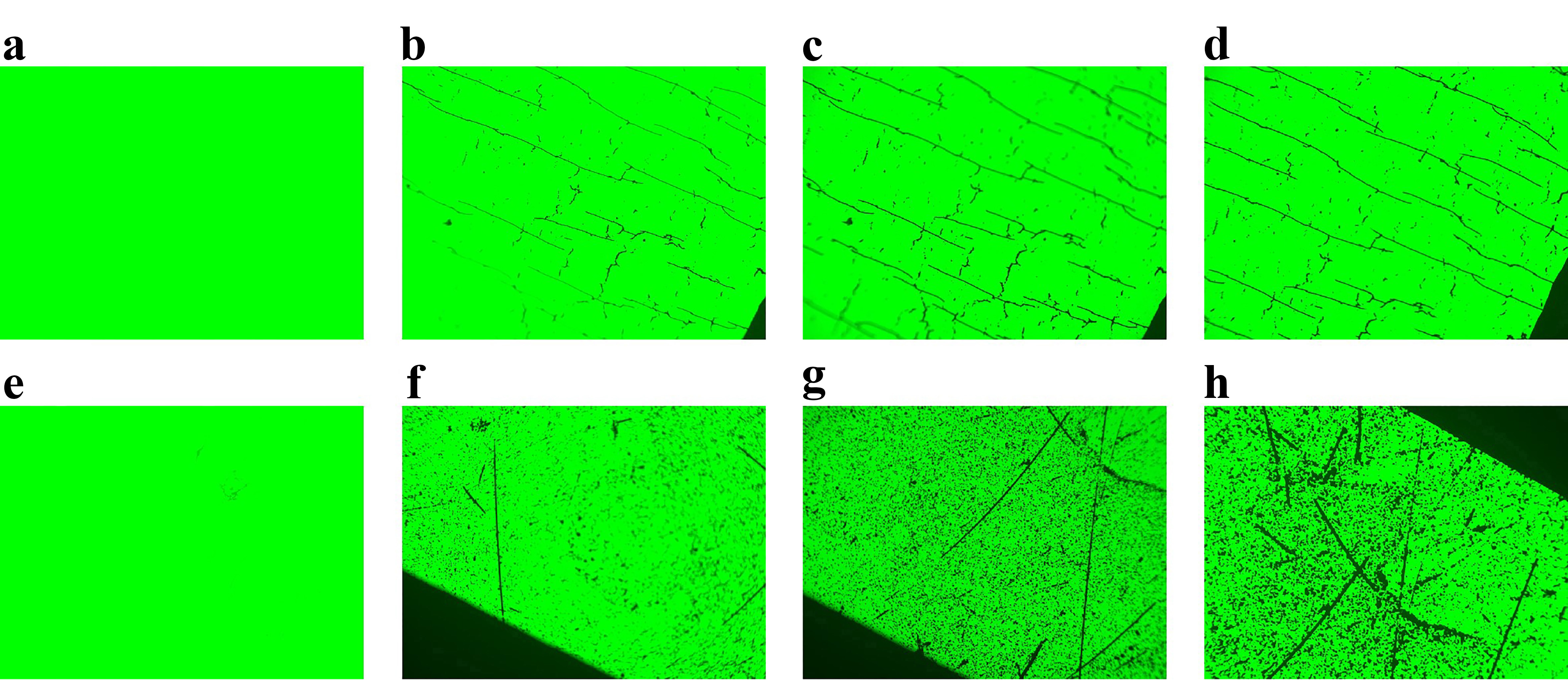


**Fig S11**. Microscopic images of paper-based OLED after bending a) 0, b) 200, c) 500, and d) 1000 times. Microscopic images of PET-based OLED after bending e) 0, f) 200, g) 500, and h) 1000 times. The bending radius is 8 mm.


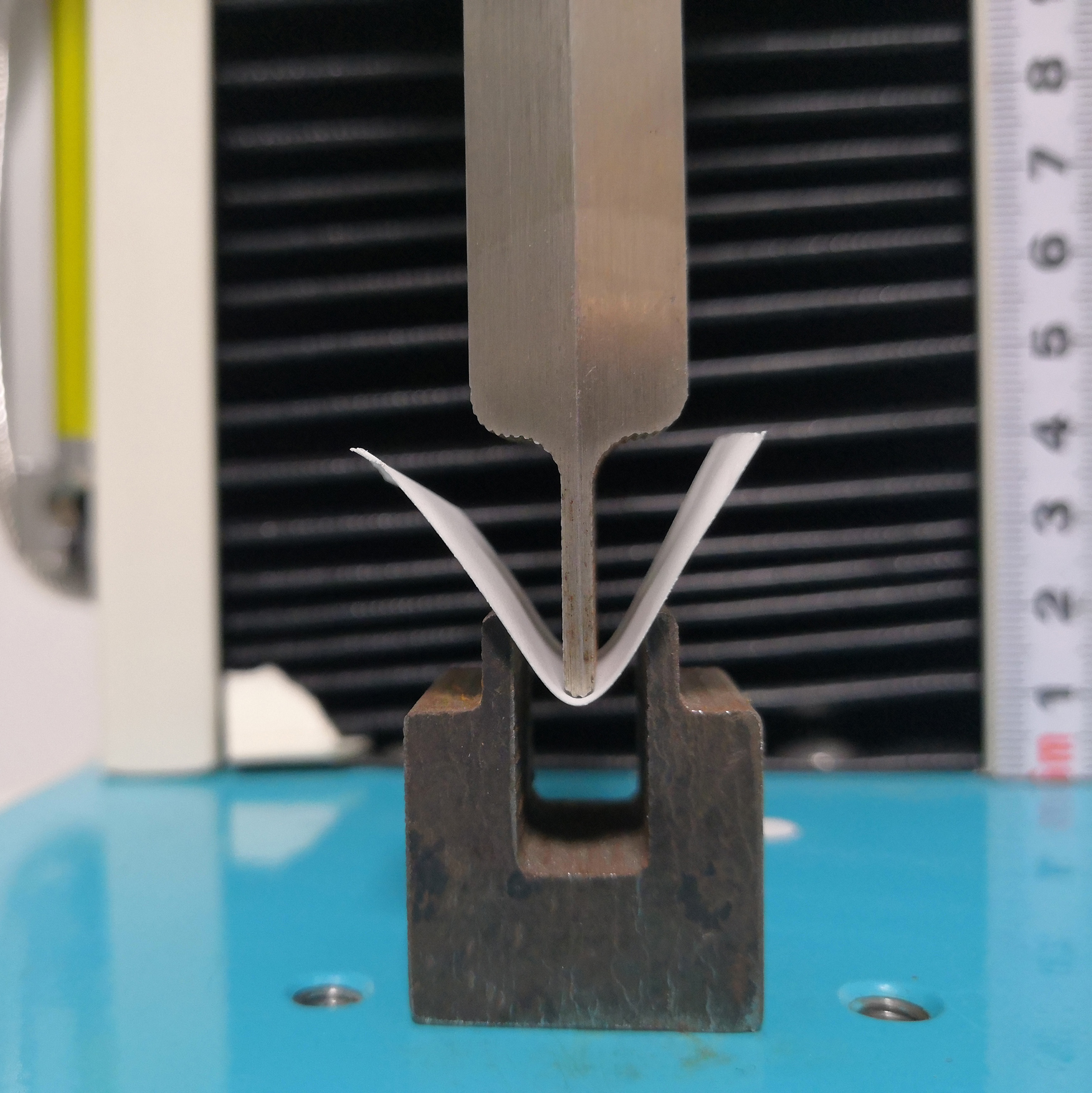


**Fig S12.** Image of the three-point bending flexural test. Effective measurement length and width of the substrate are both 10 mm.


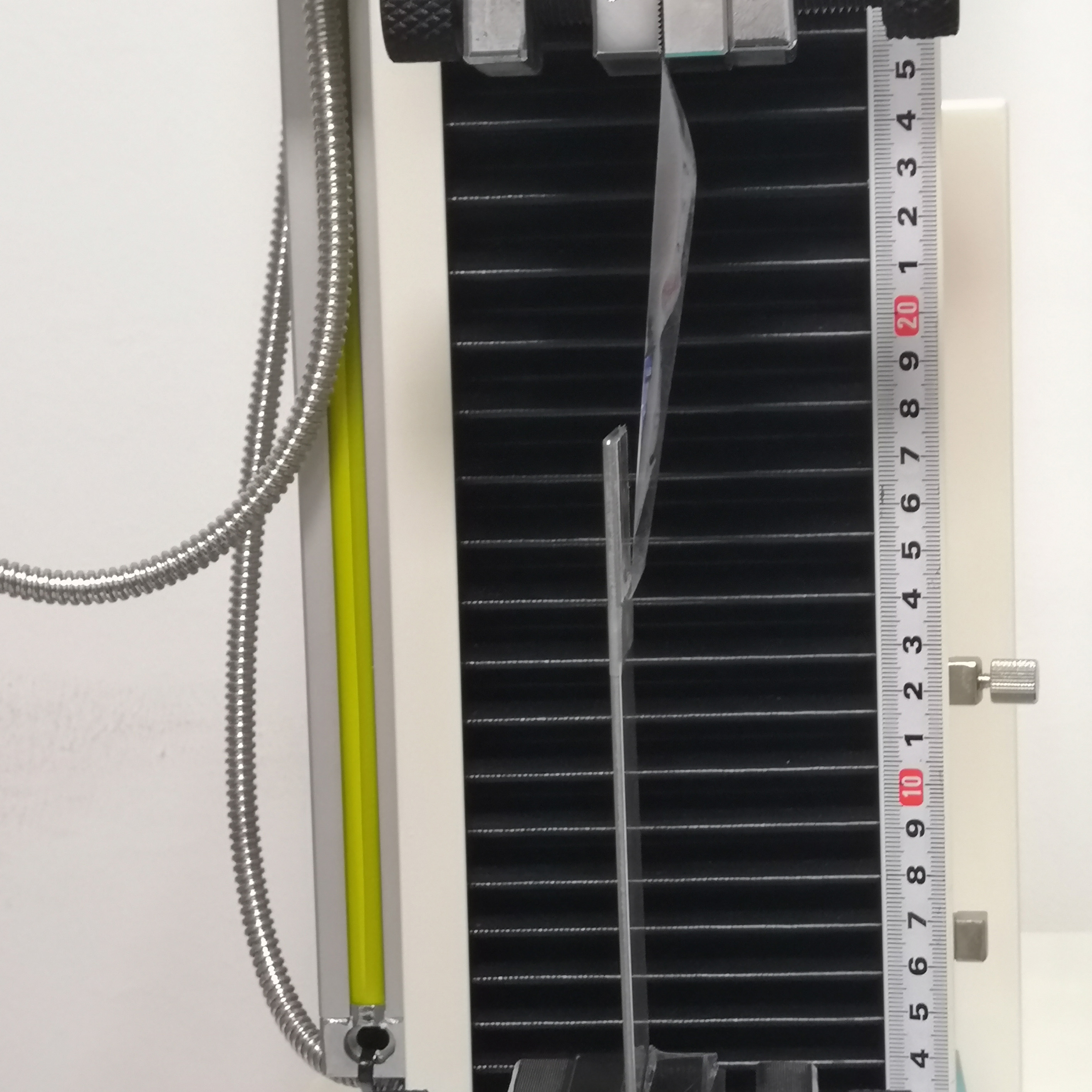


**Fig S13.** Image of the peel adhesion test.


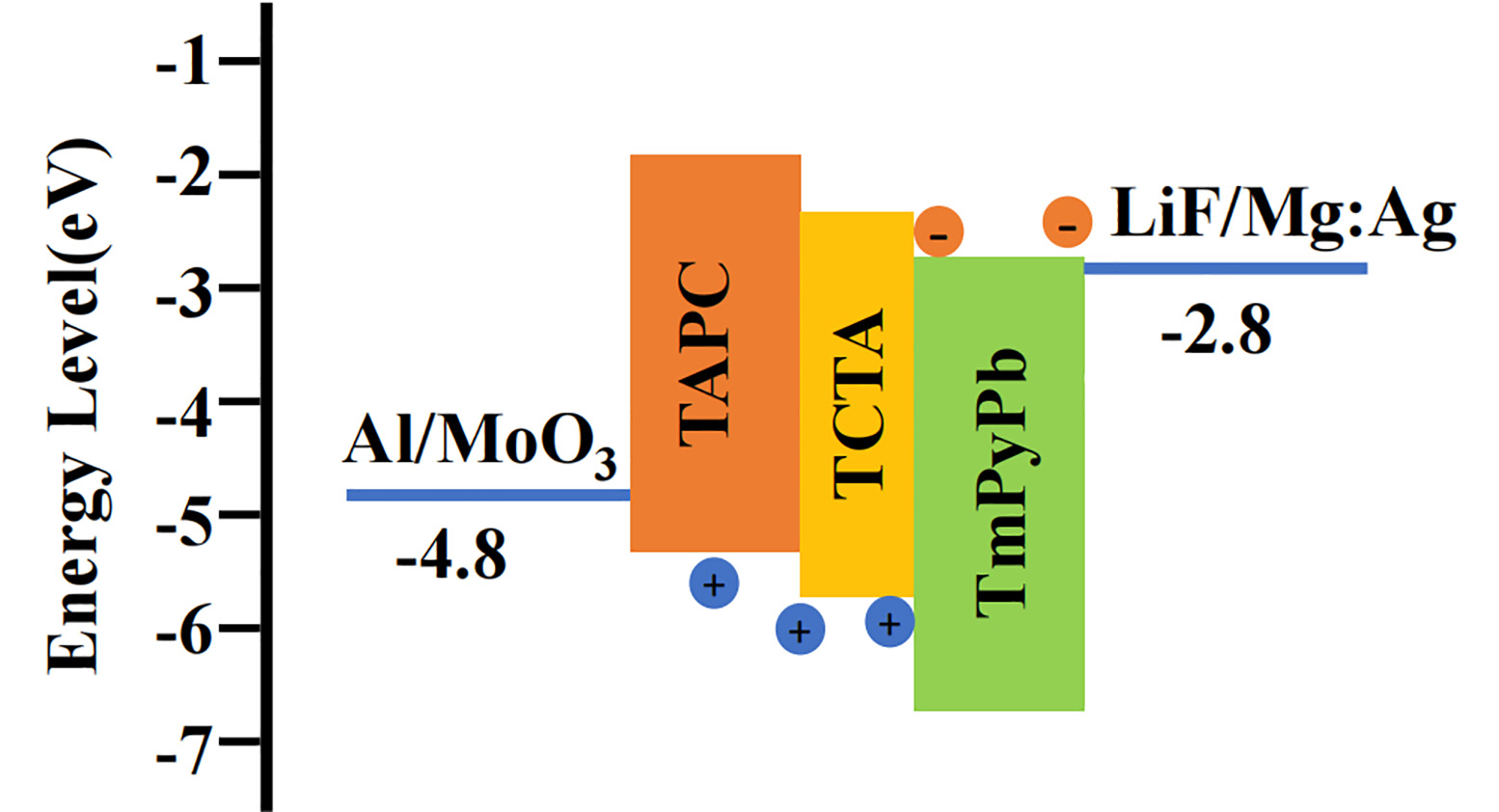


**Fig S14.** Schematic diagrams of energy levels of device background.


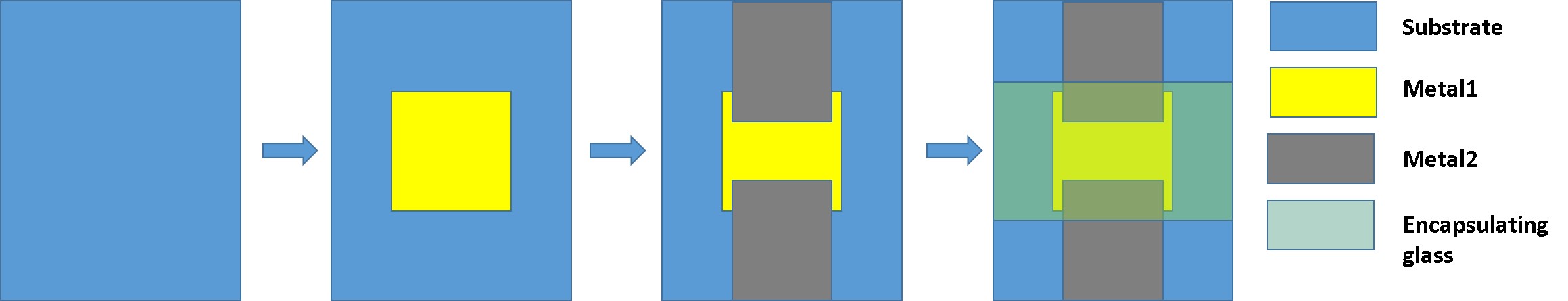


**Fig S15.** Schematic diagram of devices with the PET and the treated paper substrates for moisture and oxygen permeability experiment.


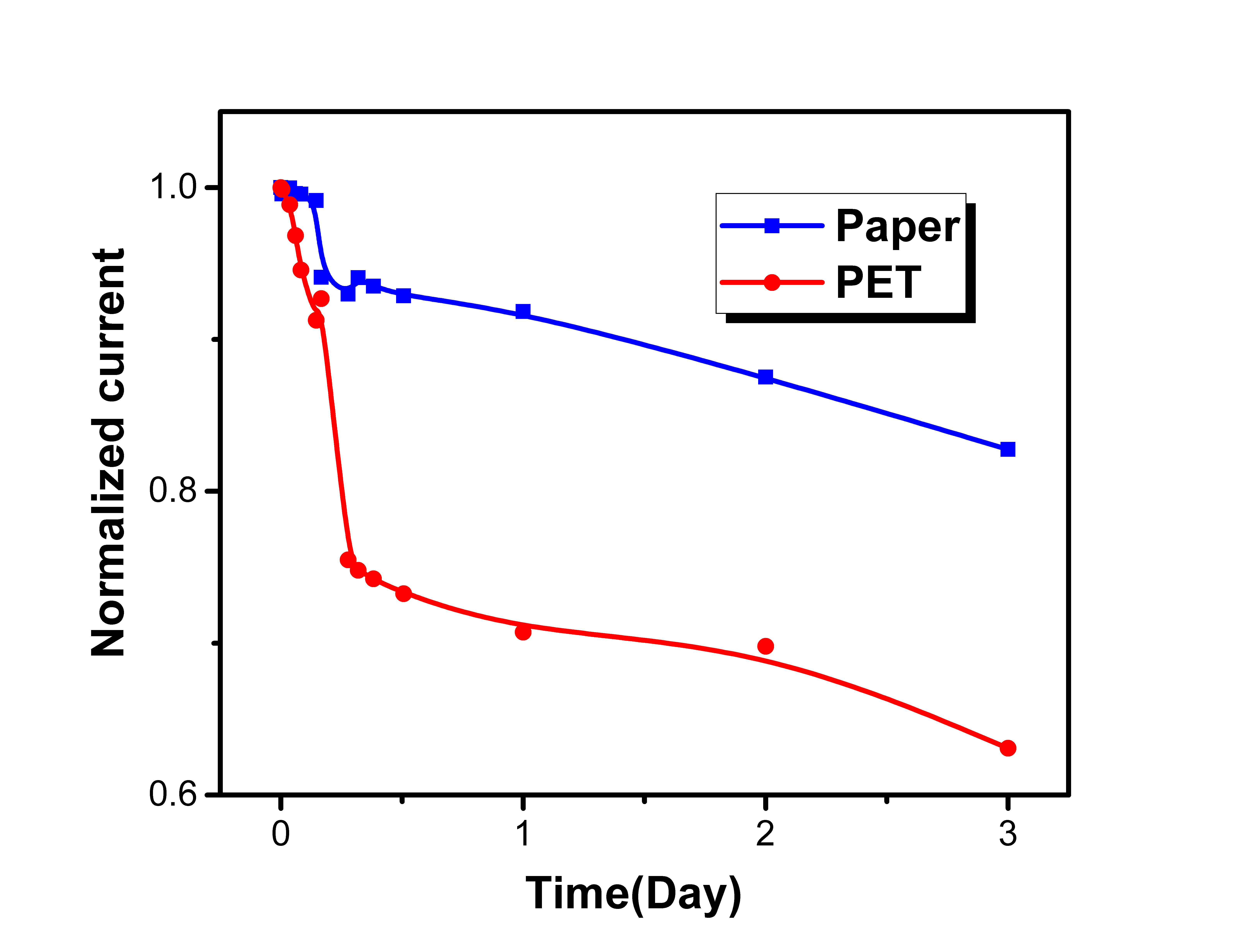


**Fig S16.** Variations of current at 2 V of devices in the moisture and oxygen permeability experiment.


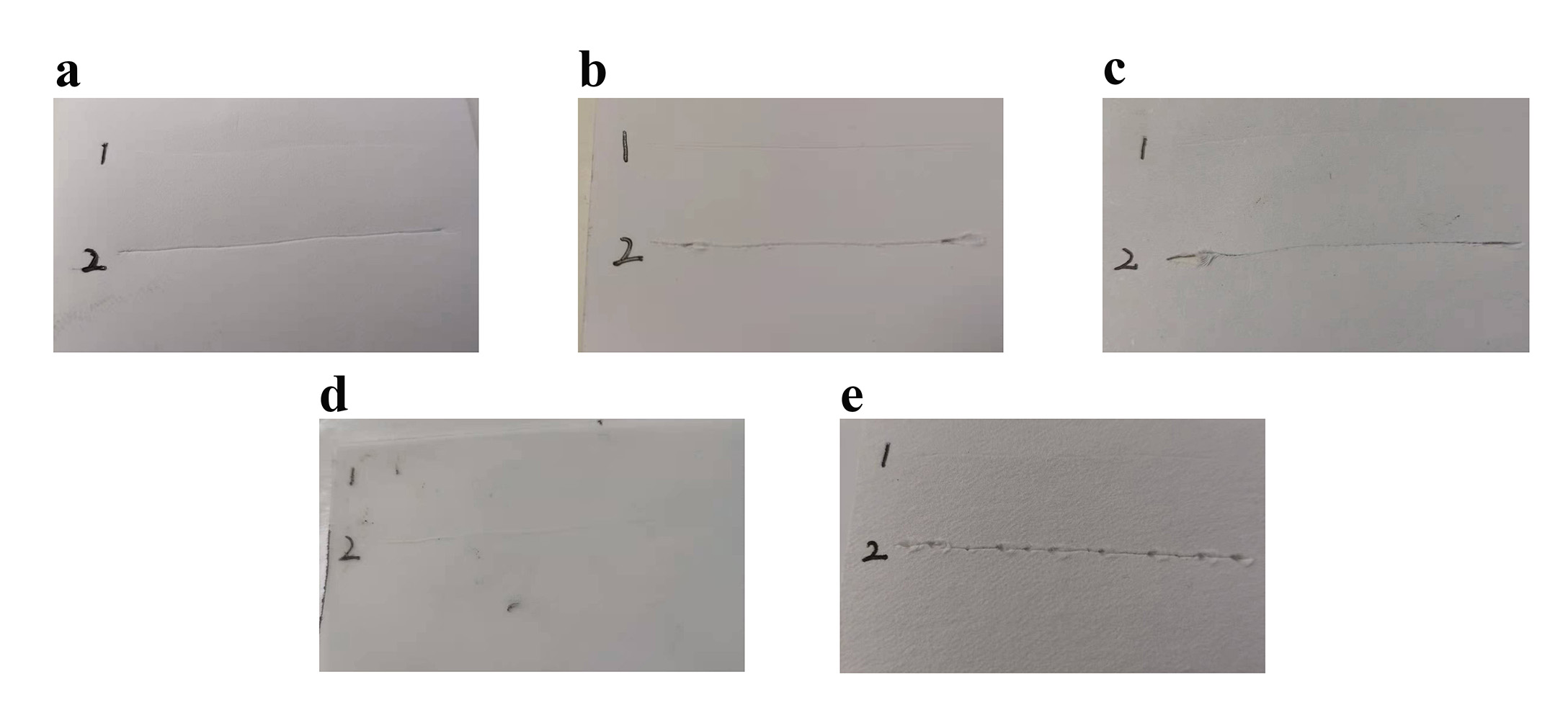


**Fig S17.** Test against scratches of a) stone paper, b) art paper, c) print paper, d) sulfuric paper and e) filter paper. Scratches marked as 1 are scratched by using a plastic tweezer, and those marked as 2 are scratched by using a metal tweezer.


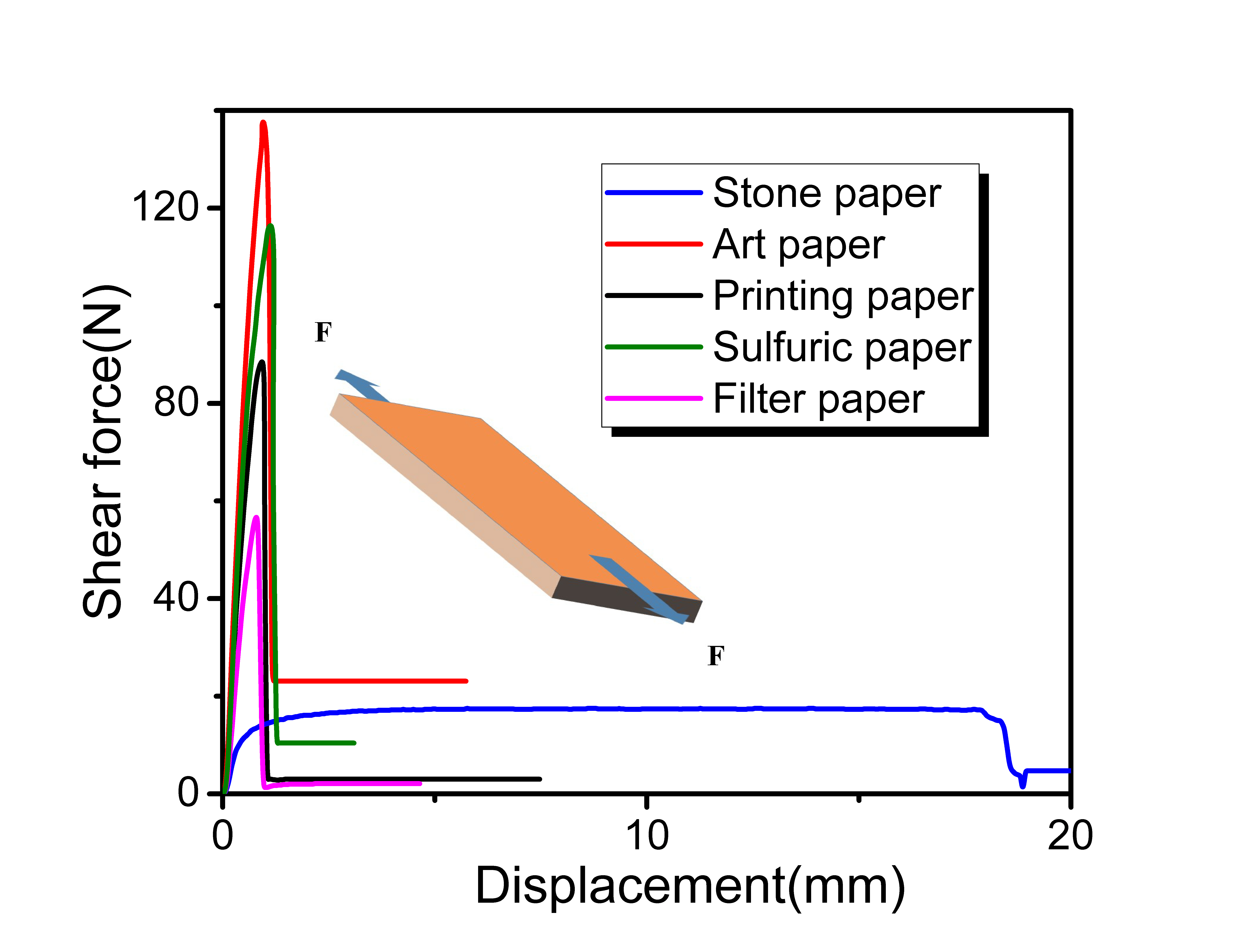


**Fig S18.** Tests against scratches test of different types of paper substrate.


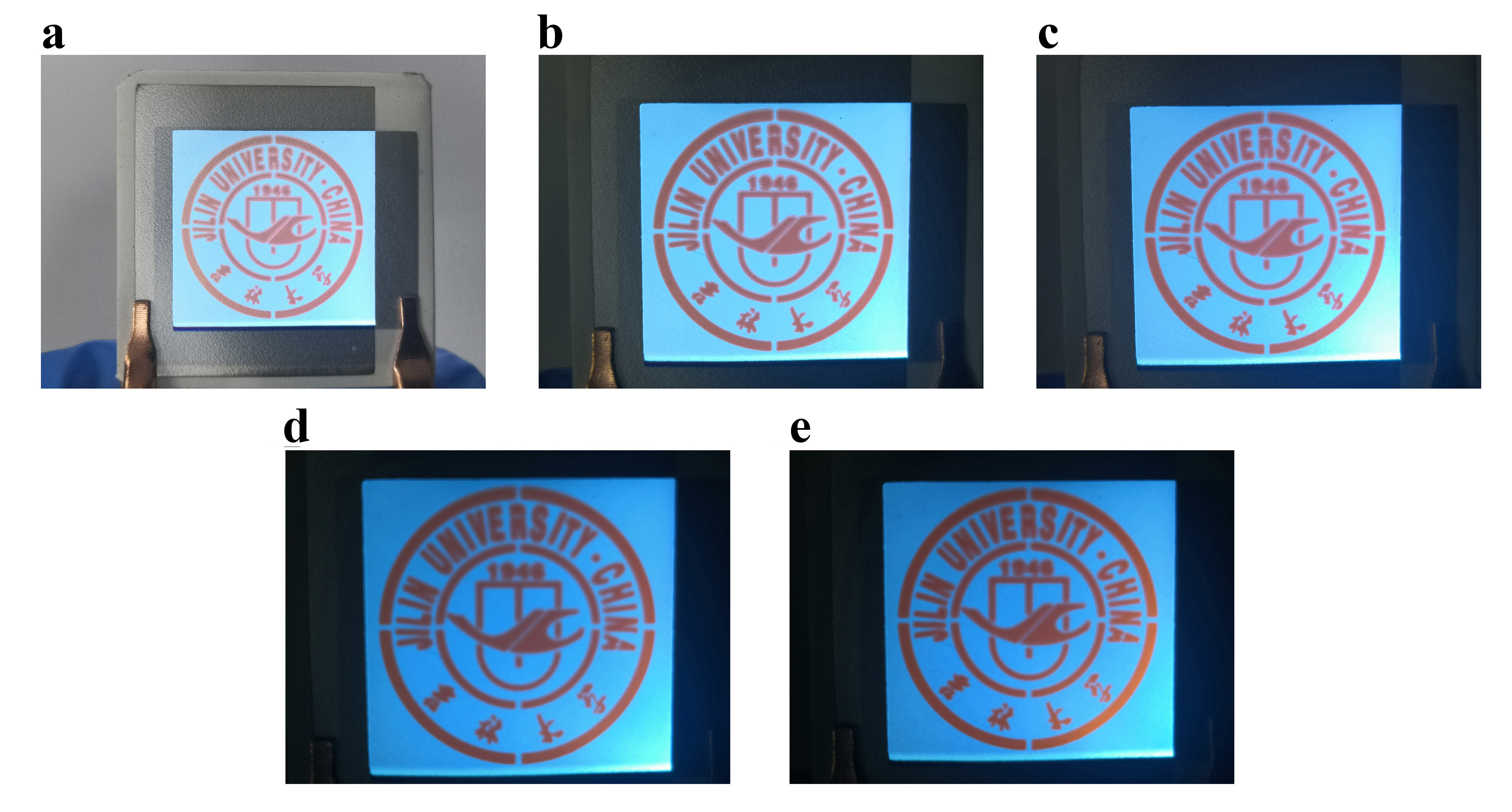


**Fig S19.** Images of white and orange FACS at different voltages: a) for 4 V, b) for 5 V, c) for 6 V, d) for 7 V, and e) for 8 V.

Table S1. Details of the structures of the devices in this work.

| Device | Anode | HIL | HTL | EBL | EML | ETL | EIL | Cathode | Cathode |
| --- | --- | --- | --- | --- | --- | --- | --- | --- | --- |
| TG-P, TG-G, TG-PET and art paper-based green device | Al  100 nm | MoO_3_  3 nm | TAPC  30 nm | TcTa  5 nm | CBP:10% Ir(ppy)3  20 nm | TmPyPB  50 nm | LiF  0.5 nm | Mg:Ag  (15:1)  1 nm | Ag  19 nm |
| Green and violet FACS | Al  100 nm | MoO_3_  3 nm | TAPC  30 nm | TcTa  5 nm | CBP:10% Ir(ppy)3  20 nm (Logo) | TmPyPB  50 nm | LiF  0.5 nm | Mg:Ag  (15:1)  1 nm | Ag  19 nm |
| Background of white and orange FACS | Al  100 nm | MoO_3_  3 nm | TAPC  30 nm |  | TcTa:10% FIrpic  10 nm  CBP:3% MDQ  1 nm | TmPyPB  40 nm | LiF  0.5 nm | Mg:Ag  (15:1)  1 nm | Ag  19 nm |
| Logo of white and orange FACS | Al  100 nm | MoO_3_  3 nm | TAPC  30 nm |  | TcTa:10% FIrpic  10 nm  CBP:3% MDQ  1 nm  CBP:3% MDQ  10 nm (Logo) | TmPyPB  40 nm | LiF  0.5 nm | Mg:Ag  (15:1)  1 nm | Ag  19 nm |

Table S2. Performances of paper-based OLEDs in reported papers and this work.

| Research | Ref 23 | Ref 24 | Ref 25 | Ref 26 | This paper |
| --- | --- | --- | --- | --- | --- |
| Maximum Brightness (cd m^-2^) | 10000 | 2200 | 2400 | 3890 | 71346 |
| Maximum Current Efficiency (cd A^-1^) | 47 | 0.7 | N/A | 5 | 64 |
| EML Material | CBP:Ir(ppy)_3_ | Alq_3_:C6 | Alq_3_ | Alq_3_ | CBP:Ir(ppy)_3_ |
| Peak Wavelength (nm) | ~515 | N/A | ~510 | N/A | ~510 |

**Table S3**. **Thickness of different substrates before and after dip-coating treatment.**

|  | Stone Paper | Art Paper | Printing Paper | Sulfuric paper | Filter paper |
| --- | --- | --- | --- | --- | --- |
| **Maximum Luminance (cd m^-2^)** | 71346 | 111159 | 19845 | 19102 | 2522 |
| **Maximum CE (cd A^-1^)** | 64.2 | 90.5 | 43.1 | 19.5 | 3.6 |

**Table S4**. **Thickness of different substrates before and after dip-coating treatment.**

|  | Stone Paper | Art Paper | Printing Paper | Sulfuric paper | Filter paper | PET |
| --- | --- | --- | --- | --- | --- | --- |
| **Thickness (**$\boldsymbol{\mu}\boldsymbol{m}$**)** | 130 | 170 | 90 | 60 | 150 | 150 |
|  | Stone Paper (dip) | Art Paper (dip) | Printing Paper (dip) | Sulfuric paper (dip) | Filter paper (dip) |  |
| **Thickness (**$\boldsymbol{\mu}\boldsymbol{m}$**)** | 160 | 210 | 120 | 80 | 170 |  |

**Table S5. Flexural Modulus and Bending Strength of different substrates.**

|  | Stone Paper | Art Paper | Printing Paper | Sulfuric paper | Filter paper | PET |
| --- | --- | --- | --- | --- | --- | --- |
| **Flexural Modulus *E_f_* (MPa)** | 1607 | 2225 | 5937 | 7969 | 7681 | 4343 |
| **Bending Strength R (MPa)** | 15.9 | 38.1 | 40.3 | 56.2 | 60.5 | 87.4 |
|  | Stone Paper (dip) | Art Paper (dip) | Printing Paper (dip) | Sulfuric paper (dip) | Filter paper (dip) |  |
| **Flexural Modulus *E_f_* (MPa)** | 844 | 4606 | 3261 | 3143 | 5603 |  |
| **Bending Strength R (MPa)** | 11.9 | 64.2 | 38.9 | 35.1 | 77.3 |  |

Note S1

The Purcell factor is given as:

$F_{P}=\frac{3}{4\pi^{2}}(\frac{\lambda_{\mathrm{free}}}{n})^{3}(\frac{Q}{V})$,

where, (*λ*_free_/*n*) is the wavelength within the cavity material of refractive index *n*, and *Q* and *V* are the quality factor and mode volume of the cavity, respectively. The *Q* decreases almost exponentially as the wavelength of emission shifts away from the resonant wavelength, resulting in a *F*_P_<1 and then an inhibited spontaneous emission rate. For devices background and logo presented in Figures 5D and 5E, their resonant wavelength at the viewing angle of 0^o^ is close to the emission wavelengths of Firpic and far from those of Ir(MDQ)_2_(acac). As the viewing angle increases, the resonant wavelength shifts away from the emission wavelengths of Firpic. Thus, compared to Ir(MDQ)_2_(acac), the spontaneous emission rate of Firpic will be inhibited more obviously. It then leads to a relatively weaker blue emission and then a red-shift.
